# Application of the data envelopment analysis technique to measure the environmental efficiency of the 27 countries of the European Union during the period 2012–2020

**DOI:** 10.1007/s10098-023-02553-9

**Published:** 2023-06-14

**Authors:** Juan Cámara-Aceituno, Manuel Jesús Hermoso-Orzáez, Julio Terrados-Cepeda, Ángel Mena-Nieto, José Enrique García-Ramos

**Affiliations:** 1 Department of Engineering Graphics, Design and Projects, University of Jaén, 23701 Jaén, Spain; 2 Center for Advanced Studies in Physics, Mathematics and Computing, University of Huelva, 21007 Huelva, Spain; 3 Department of Electrical and Thermal Engineering, Design and Projects, University of Huelva, 21071 Huelva, Spain; 4 Department of Integrated Sciences, University of Huelva, 21071 Huelva, Spain

**Keywords:** Data Envelopment Analysis (DEA), Environmental efficiency, Eco-efficiency, Environmental policy, Energy policy, Sustainable development, European Union

## Abstract

In the last decade, we have experienced a changing geopolitical context that has caused changes in the energy context. In addition, human activity contributes to global warming or sea level rising, i.e., climate change. A set of action policies have been implemented to continue fighting against this environmental situation (such as the Paris Agreement, the COP27, or the European Green Deal for 2030); therefore, it is necessary to determine whether we are on the right track. It is compulsory to develop predictive models that accurately analyze the current status and the already path undertaken. To this end, this article analyzes the environmental efficiency of the 27 countries of the European Union (excluding the UK) using the so-called data envelopment analysis (DEA). In particular, economic (GDP and GDP per capita), environmental (CO2 and CH4 emissions), electricity production data, the volume of vehicles, and the industrial production rate of the different countries were collected to calculate environmental efficiency. Once these data were collected, the environmental efficiency was calculated using two methods based on the DEA. The results show that out of the 27 countries, only 12 have a relatively high environmental efficiency, although it could be improved, implementing a set of corrections. However, other countries have a low eco-efficiency performance and they must improve in the coming years. We can highlight that rich countries are closer to achieving high environmental efficiency than less developed countries.

**Graphical Abstract:**

**Figure:**
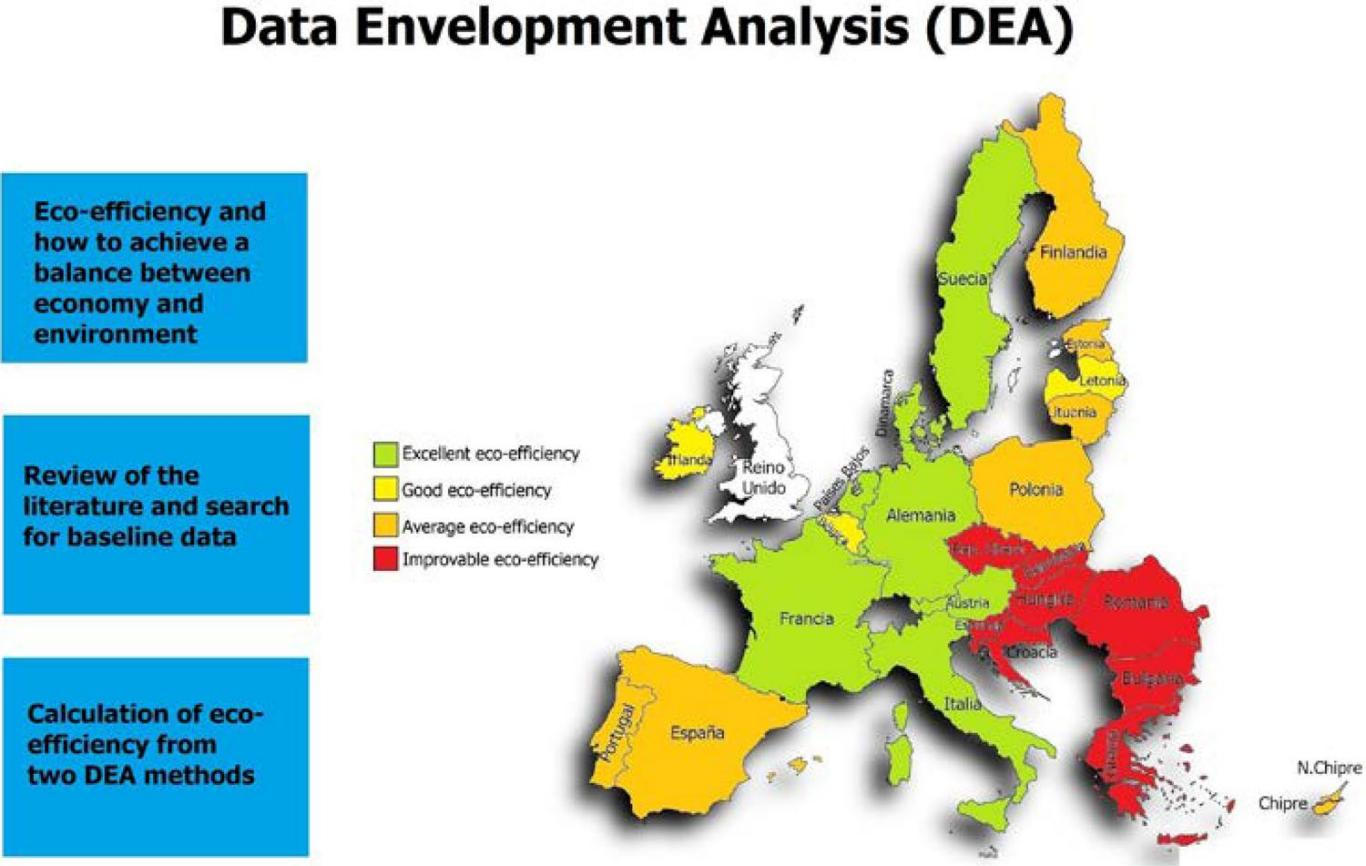
Political map of the European Union indicating the average eco-efficiency with colors of the 27 countries of the DEA method.

**Supplementary Information:**

The online version contains supplementary material available at 10.1007/s10098-023-02553-9.

## Background

In the last half million years, there have been alternating cold (glacial) and warm (interglacial) periods known as “Milankovitch cycles.” These cycles have caused the variation of the average surface temperature (15ºC) or the average value of the sea level (more than 100 m), among other climate variables. At least since the Industrial Revolution, the human activity has affected the global climate, inducing a global warming and most probably a climate change that is modifying the natural climate variability.

This global warming has accelerated significantly in recent decades, as shown by the Sixth Assessment Report (AR6) of the Intergovernmental Panel on Climate Change (IPCC) (Langsdorf et al. 2022). This report argues that, due to rapid growth of population and economic activity, carbon dioxide, methane, and nitrous oxide emissions have become more abundant than in the past 800,000 years. On the other hand, the IPCC (Masson-Delmotte et al. 2019) relates this fact with the acceleration of the global warming, namely 1.5 °C with respect to pre-industrial levels. If corrective measures are not taken, the consequences can be devastating (for example, sea level rise, which could reach 2 m by the year 2100 due to the instability of the polar caps).

To address this issue, it is necessary to take into account the economic development that different countries are experiencing since there is a close relationship between the energy use, the increase of the GDP, and the increase of greenhouse gas (GHG) emissions such as carbon dioxide (CO_2_) and methane (CH_4_). These last two, which come from industrial processes and the burning of fossil fuels, are the most important since, according to the IPCC AR6, they correspond to the 78% of the total emissions (eglitis-media 2022).

So far, most of the discussion about reducing emissions has focused on CO_2_ emissions (Ortega-Ruiz et al. 2022, 2020). However, methane has a 28 times greater impact on global warming than CO_2._ For this reason, the IPCC AR6 (Langsdorf et al. 2022) calls for paying more attention to methane emissions, which could help to mitigate the climate change and to improve the air quality worldwide. In addition, the enormous reduction in emissions that can be achieved in the transport sector by substituting the traditional fossil fuels by electricity generated with low or zero emissions should also be highlighted (Mena-Nieto et al. 2022).

The IPCC claims that, although emissions would radically diminish, global temperatures are expected to rise naturally by around 0.5ºC in the coming decades, since the emitted CO_2_ experiences a long permanence in the atmosphere. The only way to reduce this temperature increase will be by achieving a negative emissions balance.

Therefore, as it is necessary to raise awareness on this issue to reverse this situation, the European Union has already implemented policies in order to promote economic growth, taking into account the protection of the environment, guaranteeing sustainable development (Comisión Europea. Dirección General de Comunicación 2014), that is, we must ensure sustainable development.

To promote sustainable development, it has been proposed that mitigating the effect of human activity involves using renewable energy and adequately managing waste. To achieve this sustainable development, different energy policies have been established, among which are those related to the 2030 objectives (European Commission 2020; Instituto para la Diversificación y Ahorro de la Energía (IDAE) 2021) and the COP26 summit in Glasgow (United Nations 2021).

Throughout the studied period, it is necessary to highlight the European Directives most directly related to the variables included in the study. Among them, it is worth to mention the Directive 2009/28/CE (European Union 2009), which sought to reduce emissions by at least 20% compared to 1990, achieve 20% consumption of renewable energy and a 20% improvement in energy efficiency for the year 2020, and the regulation (EU) 2021/1119, called the European Green Deal (European Commission 2019; European Union 2021), which aims at reducing GHG emissions by at least 55% compared to 1990 and to be the first climate-neutral continent in the world.

Therefore, in order to achieve sustainable development, it is necessary to propose models that allow analyzing whether the path taken is the correct one or whether corrections are necessary to achieve the desired goal. This objective implies not only reducing GHG emissions, but also the economic growth must be guaranteed, and for this, it is necessary to use the term eco-efficiency (Conrad and Cassar 2014). This term is broadly used as an attempt to provide goods and services at a competitive price, meeting human needs and quality of life, while progressively reducing environmental impact and resource use, that is, designing a product that causes the least possible impact on the environment throughout its life cycle and that satisfies a defined need or can also be defined as the relationship between the value that a product provides and its environmental impact (WBCSD 2006, 2000).

Other studies established that the eco-efficiency is capable not only of relating the added value and the environmental impact of a product, but that it is s also able of being a proxy of sustainability (Oliveira et al. 2014), allowing to establish an empirical relationship with economic activities. This last definition allows us to conclude that the meaning of eco-efficiency has evolved to such an extent that it can be used at an international level (as is the case that we propose in the research) to analyze the eco-efficiency of different countries and cities or regions, through the of use of different mathematical models (Masternak-Janus and Rybaczewska-Błażejowska 2017; Wang et al. 2020; Xu et al. 2017).

It is worth to mention the works by A. Emrouznejad and M. Marra where the authors highlight eco-efficiency measurement using DEA (Emrouznejad et al. 2023) or the research carried out by S. Lueddeckens concluding that most of the studies carried out so far are deficient if one only relies on a single theoretical framework (Lueddeckens 2023). Other studies have also pointed to the usefulness of studying the impact of the economy on the sustainable development (Yin and Liu 2023). Alternatively, analyze the eco-efficiency of a specific sector (Castilho et al. 2021; Coluccia et al. 2020; Shah et al. 2020; Wang et al. 2023) only.

In order to measure eco-efficiency, many studies propose a DEA in order to identify unwanted variables. With the help of the DEA, efficiency performances can be measured by comparing several decision units (DMU) that have multiple inputs and outputs (Song et al. 2012). This method has been used in numerous fields, such as the automotive industry (Nassiri Pirbazari and Jalilian 2020), the environment (Hsieh 2022), and health sciences (Bayley et al. 2022a), among others. Eco-efficiency, over time, has acquired a high theoretical and practical value. Furthermore, with the help of DEA (Lundgren and Zhou 2017), it is possible to obtain a quite significant benefit without making assumptions regarding the relationship between the different data inputs and the different data outputs (Seiford and Thrall 1990). The objective pursued with the DEA is comparing all the decision units with the others to identify the most inefficient ones and to implement best practice scenarios.

The main aim of this research is to calculate the eco-efficiency for the 27 countries of the European Union between the period between 2012 and 2020. This project arises as a continuation of the work (Hermoso-Orzáez et al. 2020). The idea of continuing with this study is to improve the former calculations with more up-to-date data in order to focus on the present situation since the period between 2005 and 2012 does no longer describe the present situation. In addition, this study will allow us to know how the different countries have evolved and to work on forecasts that allow us to know if the decisions taken so far in politics, economy, or energy matters in the EU-27 are appropriated or not.

We intent to clearly define the different steps to implement our methodology:

- In the methodology section, we will explain the models used to calculate the eco-efficiency using the DEA method (Charnes et al. 1978) and the data used to perform the calculation. We will also do a bibliographical review to justify the reasons for the chosen variables.
- In the results section, the calculations will be presented once completed.
- Subsequently, the results will be reviewed in the discussion section, explaining why they have been obtained. Other methods will be provided, such as the improved analysis method (MAM), to help clarifying the understanding of the obtained results.
- Finally, the most relevant conclusions of the study will be presented, which will be complemented by proposed actions to be carried out by those countries whose eco-efficiency can be improved.

## Methodology

To measure eco-efficiency, it has been necessary to use a statistical method known as DEA. DEA is a nonlinear programming method developed by Charnes et al. (1978), with decision-making units (DMUs), makes possible to determine which one is more efficient. The positive aspect of the method is that it can be used in any field, such as environmental studies, the automobile industry, or health sciences, among others (Bayley et al. 2022b; Hsieh 2022; Jalilian and Pirbazari 2020).

According to Charnes et al. (1978), once the variables have been differentiated, one must determine the range of study of the considered DMUs; that is, if the 27 countries of the European Union considered in this study, we will then have 27 DMUs whose notation will be *j* = (1, 2, 3, …, 27) and for each of these DMUs we will consider *m* inputs *x*_*ij*_ (*i* = 1, 2, …, *m*) and s outputs y_*rj*_ (*r* = 1, 2, 3, …, s).

After defining each variable and its range, it will be necessary to express the nonlinear programming problem. This problem can be expressed as follows (Cook and Seiford 2009).

1 $$\begin{aligned} e_{j} & = \max \mathop \sum \limits_{r} u_{r} y_{rj} /\mathop \sum \limits_{i} v_{i} x_{ij} \\ & \quad {\text{s}}{\text{.t}}{.} \mathop \sum \limits_{r} u_{r} y_{rj} - \mathop \sum \limits_{i} v_{i} x_{ij} \le 0 \\ & \quad \quad u_{r} ,v_{i} \ge \epsilon \\ \end{aligned}$$

From the previous equation, it is identified that *ε* is a value that serves for the variables to be strictly positive. Everything else is easily identifiable, as explained above, such as *x*_*io*_ the inputs and y_*ro*_ the outputs, *i* the number of inputs, *r* the number of outputs and *u*_*r*_ and *v*_*i*_ the weights assigned to outputs and inputs, respectively.

The nonlinear programming problem is named CCR after its authors Charnes, Cooper, and Rhodes. This problem is also known as CRS (constant returns to scale).

The important thing about the model is that it can be reformulated as a linear programming model applying the fractional programming theory (Charnes and Cooper 1963) with the following changes of variables: *μ*_*r*_ = *tu*_*r*_ and ${\nu }_{i} = t{v}_{i}$_*,*_ where *t* = ${({\sum }_{i}{v}_{i}{x}_{io}) }^{-1}$. The linear model is expressed as follows:

2 $$\begin{aligned} e_{j} & = \max \mathop \sum \limits_{r} \mu_{r} y_{rj} \\ & \quad {\text{s}}{\text{.t}}{.} \mathop \sum \limits_{i} \nu_{i} x_{io} = 1 \\ & \quad \quad \mathop \sum \limits_{r} \mu_{r} y_{rj} - \mathop \sum \limits_{i} \nu_{i} x_{ij} \le 0 \\ & \quad \quad u_{r} ,\nu_{i} \ge \epsilon \\ \end{aligned}$$

The DEA method designed to calculate the environmental efficiency in this project will consist of a series of input variables (inputs) and some output variables (outputs). These output variables can be separated into two classes, desirable and undesirable, because some of them are expected to have a large value to induce a high environmental efficiency, e.g., GDP and GDP per capita, while in others is right the opposite, i.e., small values will produce a high environmental efficiency, e.g., GHG emissions. Therefore, the following inputs and desirable and undesirable outputs will be considered,

- Inputs: generation of electricity from coal, oil, or nuclear energy, the rate of industrial production, and the volume of vehicles.
- Desirable outputs: GDP and GDP per capita.
- Undesirable outputs: emissions of CO_2_ and CH_4_.

Although there are other GHGs, these were chosen because CO_2_ represents 80% of all GHG emissions in the EU, while CH_4_ is the second with 12% of the total (European Parliament 2023). In addition, according to the 2014 IPCC report, they are responsible for 78% of the emissions from 1970 to 2010 (eglitis-media 2022). The rise of these emissions clearly affects the value of eco-efficiency. Therefore, the greater the input, the greater the output, but a medium/optimal point must be found to obtain a high eco-efficiency. A bibliographic summary (Table 1) can also justify why these variables are the most appropriate for the study.

**Table 1:** Bibliographic summary of the different DEA methods with the inputs and outputs used

Investigators	Method	Study area	Inputs	Outputs
Woo et al. (2015)	DEA, Malmquist	31 OECD countries, 2004–2011	Labor, capital and energy	GDP_,_ CO_2_
Zhou et al. (2007)	Non-radial DEA, Malmquist	26 OECD countries, 1995–1997	Workforce	GDP, SO_2_, SO_x_, NO_x_, CO
Halkos and Tzeremes (2014)	Bootstrapped DEA	110countries, 2007	Labor and capital	GDP, CO_2_
Chien and Hu (2007)	DEA	45 countries, 2001 and 2002	Labor, capital and energy	GDP
Li et al. (2013)	AED Tobit	Beijing, 2005–2009	Labor, capital and energy	GDP, waste water, sold waste
Song and Guan (2014)	SESBM, Malmquist	Wanjiang demonstration area, 2010 and 2011	Population, capital and energy	GDP, industrial SO_2_
Yang et al. (2015)	HEADQUARTERS	20 provinces in China, 2000–2012	Labor, capital and energy	GDP, CO2, SO_2_
Wang et al. (2013a, b)	DEA Windows analysis	29 provinces in China, 2000–2008	Labor, capital and energy	GDP, CO2, SO_2_
Zhou et al. (2013b)	Entropy SBM DEA	30 provinces in China, 2005–2010	Labor, capital and energy	Power capacity, SO_2_, NOx, CO_2_
Yang and Wang (2013)	DEA	29 provinces in China, 2000–2007	Labor, capital and energy	GDP, CO_2_
Chang et al. (2013)	SBM DEA	30 provinces in China, 2009	Labor, capital and energy	Added value of the transport sector, CO_2_
Song et al. (2013)	SBM DEA, Tobit	29 provinces in China, 1998–2009	Labor, capital and energy	GDP, wastewater, solid waste, waste gas
Wang et al. (2013a, b)	Non-radial FDD	28 provinces in China, 2005–2010	Labor, capital and energy	GDP, CO_2_
Wang et al. (2015)	Metafrontier, DEA	211 cities in China, 2008	Labor, capital and energy	GDP, SO_2_
Zhou et al. (2013a)	Weighted SBM	27 industrial sectors in China	Investment in industry, employees, coal and gas	Industrial production, solid waste, waste gases, wastewater

The desirable outputs are those related to the economic indicators GDP (Yu et al. 2017) and GDP per capita (Sueyoshi and Yuan 2018), which provide the most accurate information on the economic situation of the country. In short, what refers to the inputs of electricity generation from coal, oil, and nuclear energy (Hermoso-Orzáez et al. 2020; Wu et al. 2016), the volume of vehicles and the rate of industrial production (Feng and Wang 2017) and of course, the outputs previously exposed, we wanted to continue using the same variables as those used in the article "Measurement of environmental efficiency in the countries of the European Union with the improved method of analysis of enveloping data (DEA) during the period 2005–2012" with the unequivocal objective of comparing periods and being able to minimize any distortion that could exist if we added other different variables. In addition, it should be clarified that the inputs are closely related to the outputs since, for example, the greater the number of combustion vehicles, the greater the emissions in the studied region. In the end, what is intended with these data is to calculate the eco-efficiency of the chosen DMUs and compare them with each other. This comparison will make it possible to identify from the energy and economic point of view (hence the importance when choosing inputs and outputs) that those countries with high eco-efficiency values are doing well and those with lower values are doing poorly. Obviously, those with low values already show that something is failing in economic-energy matters. If so, corrective measures can be proposed that allow them to develop their policies better.

Before starting to explain how the eco-efficiency is going to be calculated, we wanted to include the origin of the official data that will be used to carry out the research:

- **Eurostat:** From each country's GDP and GDP per capita data have been obtained (Eurostat 2022).
- **World Bank:** From this database, electricity generation from the different sources considered (coal, nuclear energy, and oil) has been obtained in percentages with respect to the total (World Bank 2022).
- **IPCC:** has provided the emission factors for fossil fuels that have made it possible to calculate CO_2_ emissions. In any case, the calculations have been corroborated with official data provided by Eurostat since it has also been necessary to obtain those of CH_4_ emissions (IPCC 2006).

Data sources from the countries have also been used, such as the Spanish INE or sources related to the national statistical institutes (Expansion 2022; INE 2022).

Then, we wanted to include a table with a small statistical study that summarizes the most relevant data used in the eco-efficiency calculations. In addition, the reader will also be able to see the units of each one of them (Table 2).

**Table 2:** Statistical study of entries and exits for the 27 countries of the European Union, 2012–2020

	Indicator	Unit	Maximum	Minimum	Average	Typical deviation
Inputs	Generation of electricity from coal	%	85.206	0	17.529	19.323
	Generation of electricity from nuclear energy	%	78.235	0	16.928	21.588
	Generation of electricity from oil	%	98.866	0	6.905	21.304
	Industrial production rate	%	36.725	-13.375	1.174	4.968
	traffic volume	Vehicles	53,651,934	297,002	9,976,265.21	14,293,763.86
Desirable outputs	GDP	Millions of €	3,479,367	7,364.5	467,277.33	740,413.96
	GDP per capita	€	101,760	5,780	28,247.94	19,255.10
Undesirable outputs	CO_2_ emissions	Kts	823,068.6	1,345.63	112,505.09	165,624.91
	CH_4_ emissions	Kt of CO _2_	64,940.00	189.999	13,939.3927	16,469.27152

It should be noted that the electricity generation data have been taken in percentages because they are expressed over the total electricity produced in each country. In contrast, the rate of industrial production, which is also a percentage, has been calculated as the average of the 12 values obtained per year for the same country. The vehicles refer to those that run on fossil fuels in each of the chosen countries.

Observing Table 2 and, more specifically, the data for electricity generation from coal, Poland is the country that uses this source in the most significant proportion to cover electricity demand, in 2013 when it reached a maximum of 85%. The minimum is marked by Cyprus, Estonia, Latvia, Lithuania, Luxembourg and Malta, which did not produce electricity from coal. Malta monopolizes electricity generation from oil (until 2015) and Cyprus, producing up to 98% and 90% of electricity from that source, respectively. The minimum is set by Luxembourg and Estonia, with electricity production from oil very close to 0% in some years. Regarding the obtaining of electricity from nuclear energy, France is the country that uses this source the most to cover its electricity demand, around 70–80%. Many other countries do not use this energy to produce electricity, such as Austria, Cyprus, Croatia, Denmark, Estonia, Greece, Ireland, Italy, Latvia, Lithuania, Luxembourg, Malta, Poland and Portugal.

Germany is the country with the highest number of vehicles, and Malta is the country with the lowest number of them. Regarding the industrial production rate, Ireland is the one that presents the maximum value, while Cyprus presented the minimum value in 2013.

Regarding the economic indicators that will be used as desired results, the highest GDP in the EU-27 is Germany, with some 3,479,367 M€ in 2019, and the lowest is Malta, with a minimum of 73,645 M€ in 2012. On the other hand, the highest GDP per capita is presented by Luxembourg, with a maximum value of €101,760 in 2020, and the lowest value corresponds to Bulgaria, with €5,780 in 2012.

To finish with emissions, the country with the highest CO_2_ emissions is Germany (823,069 kton), and Malta the country with the least (1,346 kts). France has the highest CH4 emissions, with a maximum value of 64,940 kt CO2 equivalent in 2012, and Malta, with a minimum value in 2013 of 190 kt CO2 equivalent.

Once this small statistical study has been carried out, the environmental efficiency will be calculated using the DEA method. For this, it has been decided to use the two DEA methods described by Lundgren and Zhou (Lundgren and Zhou 2017), which we will call method 1 and method 2 from now on. Using two methods is a way of trying to get the results obtained to converge from a different approach and choosing which of the two may be more appropriate when it comes to eco-efficiency.

### Method 1

Starting from what has been explained previously, it is assumed that 27 countries correspond to 27 DMUs and that for each DMU, there will have “*n*” inputs and “*m*” desirable outputs, and “*j*” undesirable outputs. The notation of the DMUs will be done with the variable *k* (*k* = 1, 2, 3 … K). Once the number of inputs and outputs has been defined, it will be necessary to pose the problem by imposing a series of conditions as shown below (Tyteca 1997):

3 $$\begin{aligned} EEI & = \min \lambda \\ & \quad {\text{s}}{\text{.t}}{.}\quad \mathop \sum \limits_{k = 1}^{K} z_{k} x_{nk} \le x_{{nk^{\prime } }} , \quad n = 1, 2, \ldots ,N\quad {\text{where}}\,k^{\prime } = 1,2 \ldots K \\ & \quad \mathop \sum \limits_{k = 1}^{K} z_{k} y_{mk} \ge y_{{mk^{\prime } }} ,\quad m = 1, 2, \ldots ,M\quad {\text{where}}\,k^{\prime } = 1,2 \ldots K \\ & \quad \mathop \sum \limits_{k = 1}^{K} z_{k} u_{jk} = \lambda u_{{jk^{\prime } }} ,\quad j = 1, 2, \ldots ,J\quad {\text{where}} \,k^{\prime } = 1,2 \ldots K \\ & \quad z_{k} \ge 0,\quad k = 1,2, \ldots ,K \\ \end{aligned}$$

From the above equation, we have:

- *EEI:* refers to the environmental efficiency index (Environmental Efficiency Index), calculated by minimizing the lambda parameter.
- *Z*_*k*_*:* is the efficiency index of each country that will be varied until finding a solution that can satisfy all the conditions.
- *X*_*n*_*:* are the available inputs, and “*n*” is the number of inputs.
- *Y*_*m*_*:* are the desirable outputs available, and “*m*” is the number of desirable outputs.
- *U*_*jk*_*:* are the undesirable outputs, and “*j*” is the number of undesirable outputs.

Observing Eq. 3, we are going to comment on the following conditions:

- In the first condition, the sum of the product of the efficiency of the countries and the inputs must be less than or equal to the input of the country under study.
- The second condition considers that the sum product of the efficiency of each country and its desirable outputs must be greater than or equal to the desired outputs of the country under study.
- The third condition establishes that the sum product of the efficiency of the countries and the undesirable outputs must be equal to the product of the lambda parameter with the undesirable output of the country studied, always looking for this to be the minimum.
- The efficiency of the countries must, of course, be greater than or equal to 0.

The way to proceed will be the one described in the example made by Cook and Zhu in chapter 2 of the book *Data Envelopment Analysis: Modeling Operational Processes and Measuring Productivity* (Liang et al. 2008). With the help of the “Excel” software, the conditions described above can be imposed using the “Solver” tool for each of the countries, and, therefore, the eco-efficiency of each of them will be obtained.

### Method 2

In this method, we will proceed in the same way as in the first, but the weights of the undesirable outputs will be changed since methane emissions (CH_4_) will be considered more contributors to global warming than those of CO_2_.

The method that gave different importance to these outputs was the one proposed by Lundgren and Zhou (2017) since it defined that some variables were more important than others. The method is described as follows:

4 $$\begin{aligned} EEI & = \min \mathop \sum \limits_{j = 1}^{J} w_{j} \lambda_{j} \\ & \quad {\text{s}}{\text{.t}}{.}\quad \mathop \sum \limits_{k = 1}^{K} z_{k} x_{nk} \le x_{{nk^{\prime } }} ,\quad n = 1, 2, \ldots ,N\quad {\text{where}}\quad k^{\prime } = 1,2 \ldots K \\ & \quad \mathop \sum \limits_{k = 1}^{K} z_{k} y_{mk} \ge y_{{mk^{\prime } }} ,\quad m = 1, 2, \ldots ,M\quad {\text{where}}\quad k^{\prime } = 1,2 \ldots K \\ & \quad \mathop \sum \limits_{k = 1}^{K} z_{k} u_{jk} = \lambda_{j} u_{{jk^{\prime } }} ,\quad j = 1, 2, \ldots ,J\quad {\text{where}}\quad k^{\prime } = 1,2 \ldots K \\ & \quad z_{k} \ge 0, \lambda_{j} \le 1 \quad k = 1, 2, \ldots ,K\quad j = 1,2, \ldots ,J \\ \end{aligned}$$

The difference with method 1 is that in this case, we have “*w*_j_” which refers to the weights of the undesirable outputs.

The procedure is the same as with method 1 but adding weights of 2.86% to CO_2_ and 97.2% to CH_4._ In this method, more weight has been given to CH_4_ emissions than to CO_2_ emissions because methane contributes much more to global warming than carbon dioxide. Although CO_2_ is considered the focus of polluting emissions, it turns out that methane is about 28 times more powerful as a gas that retains heat to a greater extent (Cain et al. 2019).

## Results

Once all the calculations have been made by both methods, the results will be presented to obtain the pertinent conclusions and propose alternatives if necessary.

### Results Method 1

The results obtained by method 1 are shown year by year in Table 3

**Table 3:** Environmental efficiency results of the 27 countries of the European Union in the period 2012–2020 obtained by method 1

Method 1	2012	2013	2014	2015	2016	2017	2018	2019	2020
Austria	1	1	1	1	1	1	1	1	1
Belgium	0.7197	0.7519	1	1	1	1	1	1	1
Bulgaria	0.216	0.2193	0.1962	0.1804	0.1961	0.2082	0.1587	0.1705	0.1972
Croatia	1	0.5579	0.5355	0.5205	0.4915	10,000	0.6177	0.4792	0.4416
Cyprus	1	1	0.6963	0.5309	0.4405	0.4776	0.505	0.539	1
Czech Republic	0.2156	0.1786	0.1788	0.1543	0.1663	0.1697	0.1958	0.201	0.182
Denmark	1	1	1	1	1	1	1	1	1
Estonia	1	1	1	1	1	0.5301	0.3064	1	0.9304
Finland	0.56	0.5304	1	1	0.4899	0.8045	0.5452	0.5653	0.5081
France	1	1	1	1	1	1	1	1	1
Germany	1	1	1	1	1	1	1	1	1
Greece	0.4545	0.5545	1	0.4182	0.413	0.3922	0.3927	0.3904	0.3597
Hungary	0.3322	0.3253	0.3193	0.2675	0.282	0.2678	0.2787	0.2874	0.2395
Ireland	1	1	1	1	1	1	1	1	1
Italy	1	1	1	1	1	1	1	1	1
Latvia	1	1	1	1	1	1	1	1	1
Lithuania	1	1	1	1	1	1	1	1	1
Luxembourg	1	1	1	1	1	1	1	1	1
Malta	1	1	1	1	1	1	1	1	1
Netherlands	1	1	1	1	1	1	1	1	1
Poland	1	1	1	1	1	1	1	1	1
Portugal	1	0.5672	0.5032	0.4296	0.4514	0.4175	0.4818	1	1
Romania	0.2404	0.287	0.2881	0.2687	0.2828	0.2594	0.2665	1	1
Slovakia	0.3691	0.35	0.3657	0.3302	0.3274	0.451	0.2476	0.2845	0.2965
Slovenia	0.604	0.6113	0.8624	0.6258	0.5804	0.5773	0.5533	0.605	0.5736
Spain	1	0.7759	0.7406	0.717	0.7361	0.7094	0.7219	0.7428	0.7252
Sweden	1	1	1	1	1	1	1	1	1

### Results Method 2

Once the results of method 1 have been presented, they will be transferred to the results obtained by method 2. This method gives more weight to CH4 emissions than CO2 emissions, as previously stated.

The results calculated for environmental efficiency by this method are shown for all years in Table 4

**Table 4:** Environmental efficiency results of the 27 countries of the European Union in the period 2012–2020 obtained by method 2

Method 2	2012	2013	2014	2015	2016	2017	2018	2019	2020
Austria	1	1	1	1	1	1	1	1	1
Belgium	0.6163	0.7016	0.572	1	0.9397	1	1	1	0.4563
Bulgaria	0.0747	0.0788	0.1738	0.0784	0.0815	0.0812	0.0794	0.0845	0.0824
Croatia	0.3231	0.1628	0.457	0.1576	0.158	10,000	0.158	0.148	0.1549
Cyprus	1	1	0.3969	0.3871	0.3882	0.3842	0.3836	0.385	0.6331
Czech Republic	0.17	0.1532	0.1482	0.1489	0.1542	0.1637	0.1737	0.1788	0.1515
Denmark	1	1	1	1	1	1	1	1	1
Estonia	0.4431	0.4601	0.467	1	0.4974	0.4905	0.2827	0.4402	0.4758
Finland	0.4816	0.4738	1	1	0.4725	0.8015	0.5032	0.5108	0.4326
France	1	1	1	1	1	1	1	1	1
Germany	1	1	1	1	1	1	1	1	1
Greece	0.4098	0.43	10,000	0.3975	0.3395	0.3582	0.3176	0.3102	0.291
Hungary	0.1704	0.1693	0.1736	0.1739	0.1735	0.1857	0.195	0.2037	0.1674
Ireland	0.3378	1	1	1	1	1	1	1	1
Italy	1	1	1	1	1	1	1	1	1
Latvia	1	0.3011	1	0.3014	1	1	1	1	0.3127
Lithuania	0.1587	0.1664	1	0.1681	0.1725	0.1511	0.1545	0.1625	0.1651
Luxembourg	1	1	1	1	1	1	1	1	1
Malta	1	1	1	1	1	1	1	1	1
Netherlands	1	1	1	1	1	1	1	1	1
Poland	1	1	1	0.3506	0.3304	0.357	0.3828	0.3893	0.4301
Portugal	1	0.3319	0.3551	0.3152	0.3139	0.3893	0.3577	0.3455	0.3006
Romania	0.1035	0.1061	0.1079	0.1065	0.1107	0.1178	0.1266	1	0.1317
Slovakia	0.2238	0.2074	0.2342	0.2084	0.1999	0.449	0.2072	0.2037	0.1835
Slovenia	0.3064	0.3179	0.7734	0.3244	0.3178	0.3255	0.3077	0.3262	0.3305
Spain	1	0.5202	0.4934	0.4638	0.4596	0.4956	0.4529	0.6112	0.6385
Sweden	1	1	1	1	1	1	1	1	1

### General results of both methods

The average values for the entire period (2012–2020) for each of the EU-27 countries have also been obtained below (Table 5).

**Table 5:** Mean values of methods 1 and 2 to obtain the mean environmental efficiency for the 27 countries of the European Union for the period 2012–2020

	Method 1	Method 2	Average
Austria	1	1	1
Belgium	0.9413	0.81	0.8757
Bulgaria	0.1936	0.0909	0.1423
Croatia	0.6271	0.303	0.465
Cyprus	0.6877	0.5498	0.6187
Czech Republic	0.1824	0.1604	0.1714
Denmark	1	1	1
Estonia	0.863	0.5084	0.6857
Finland	0.6671	0.6293	0.6482
France	1	1	1
Germany	1	1	1
Greece	0.4861	0.4275	0.4568
Hungary	0.2889	0.1798	0.2343
Ireland	1	0.9262	0.9631
Italy	1	1	1
Latvia	1	0.7683	0.8842
Lithuania	1	0.2587	0.6293
Luxembourg	1	1	1
Malta	1	1	1
Netherlands	1	1	1
Poland	1	0.5824	0.7912
Portugal	0.6501	0.4125	0.5313
Romania	0.4326	0.212	0.3223
Slovakia	0.3358	0.2357	0.2857
Slovenia	0.6215	0.3705	0.496
Spain	0.7632	0.57	0.6666
Sweden	1	1	1

In addition, the eco-efficiency for the whole of the EU-27 has also been calculated by both methods, and the average is calculated in Table 6.

**Table 6:** Average environmental efficiency values of the European Union in the period studied between 2012 and 2020

	Method 1	Method 2	Average	Average Total European Union
2012	0.8041	0.6600	0.7321	0.6964
2013	0.7670	0.6141	0.6906	
2014	0.8032	0.7168	0.7600	
2015	0.7572	0.6141	0.6857	
2016	0.7355	0.5966	0.6661	
2017	0.7505	0.6574	0.7040	
2018	0.7138	0.5956	0.6547	
2019	0.7876	0.6407	0.7142	
2020	0.7946	0.5681	0.6813	

## Discussion

Next, the results obtained by both methods for each of the countries and for each of the years of the period studied will be analyzed. It should be noted that efficiency is measured from 0 to 1 (from less to more efficient); that is, those countries that present a value closer to 1 will be more efficient than the others, but this does not mean that their efficiency is largest possible, but rather, that they are the most efficient of the set of countries studied (relative environmental efficiency).

Once this comparison has been made, we will rely on a graphical representation with color maps to clarify environmental efficiency across the European Union. Once this is done, the average of both methods for all countries will be used to obtain more general results and further facilitate the discussion.

Finally, a comparison will be made between the current study period (2012–2020) with the study from 2005 to 2012 to understand how the situation has evolved over the decade. Throughout the process, we have found certain peculiarities that require a deeper analysis, which is why we also apply an improved analysis method (MAM) to help give more clarity to the investigation.

### Classification criteria for eco-efficiency: comparison between both methods.

One way to decide which countries are more eco-efficient than others is through a simple and practical classification. For this, we will use the criteria used in the previous study, "Measuring environmental efficiency in the countries of the European Union with the improved method of data environment analysis (DEA) during the period 2005–2012" since it is consistent with the method made and was accepted at the time.

The classification criteria are the following:

- Excellent eco-efficiency: all values between 0.9999 and 1. They will be assigned a green color.
- Good eco-efficiency: range between 0.8 and 0.9999. It will be assigned a yellow color.
- Medium eco-efficiency: range between 0.5 and 0.79, represented in orange.
- Poor or improvable eco-efficiency: with red color and a range between 0 and 0.49.

Once the classification was done, we wanted to use a table with colors to make the classification of the countries by both methods even more visual (Table 7). In addition to the table, we wanted to make use of a map of Europe to be able to represent the countries with the colors of the category to which they belong by both methods.

**Table 7:**
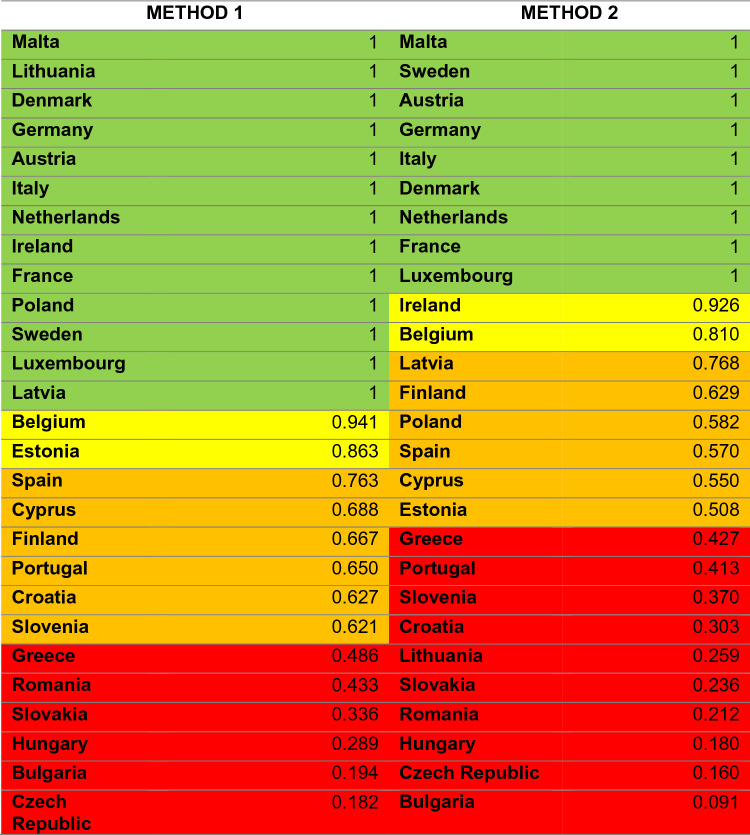
Comparison of the average environmental efficiency of both methods for the 27 countries in the period 2012–2020

**Fig. 1:**
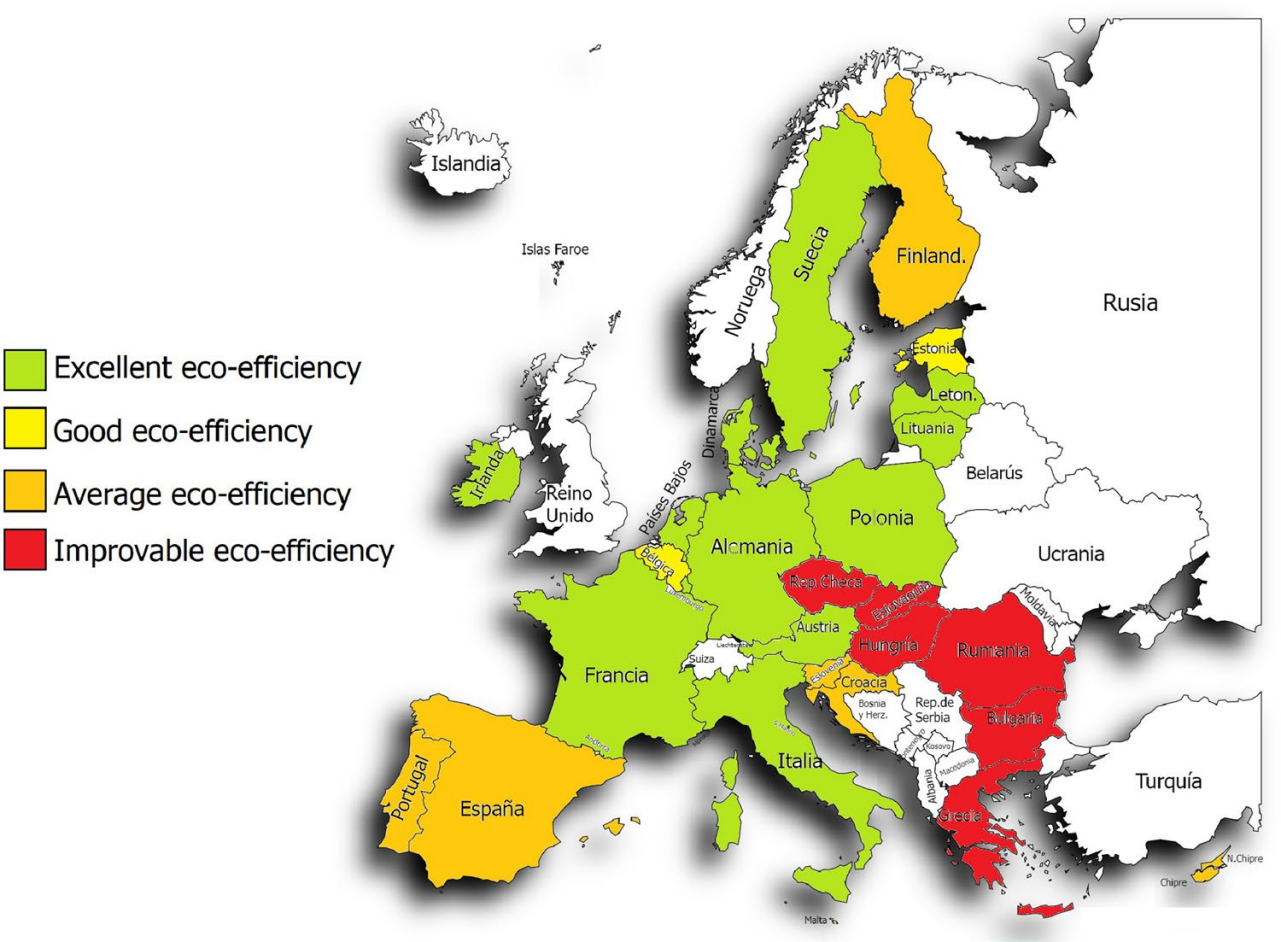
Political map of the European Union indicating the eco-efficiency with colors of the 27 countries by method 1 DEA

**Fig. 2:**
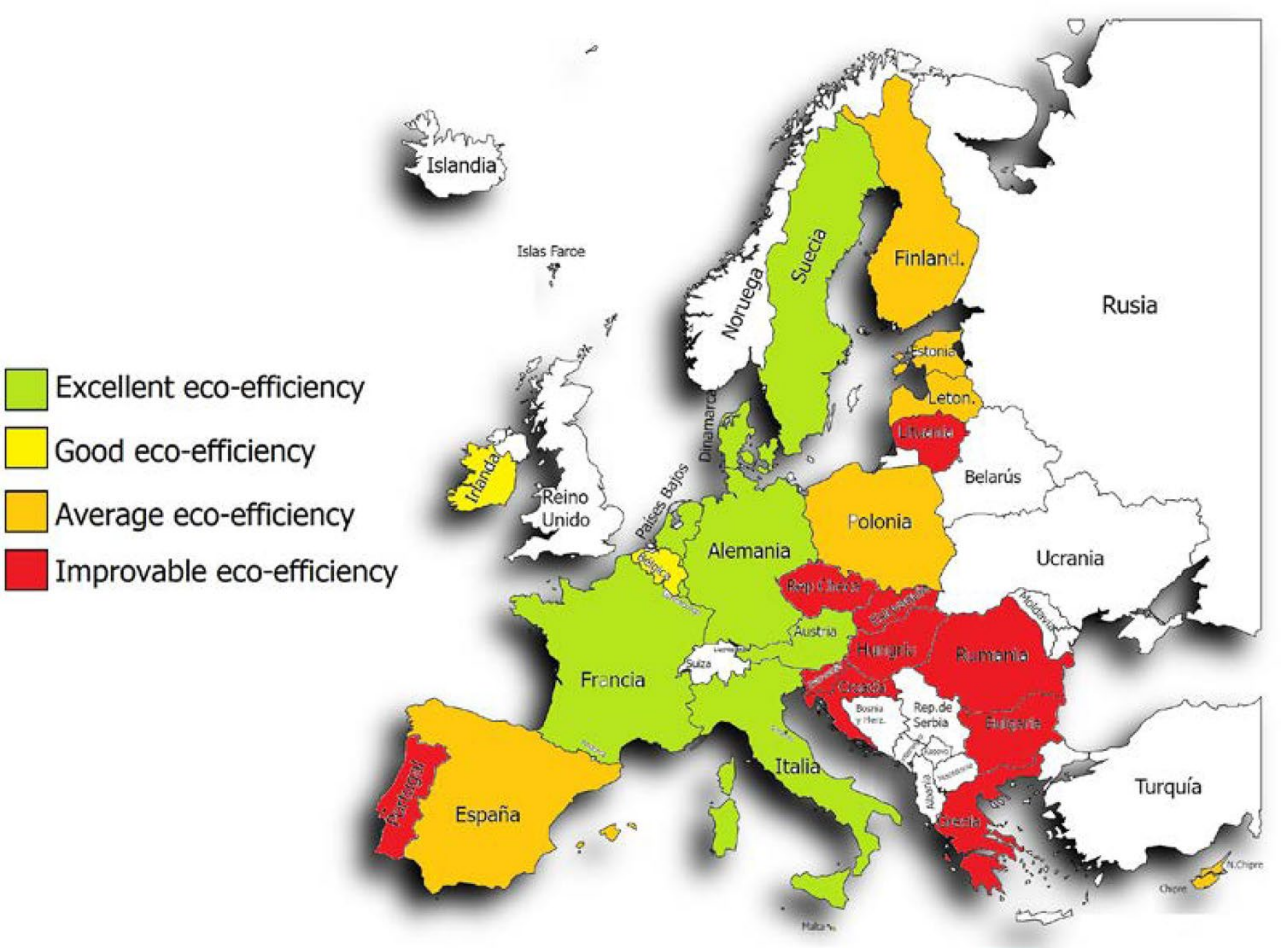
Political map of the European Union indicating the eco-efficiency with colors of the 27 countries by method 2 DEA

If we look closely (Fig. 1) we can see that the countries with the best environmental efficiency are those located in the center of Europe, while those with poor or medium efficiency are located more in the southeast of Europe. As for the countries that present medium and good environmental efficiency, they are distributed in a less uniform way since they are distributed in the periphery and one or the other more in the center. With method 2 (Fig. 2), it can be reaffirmed that the trend is very similar to that of method 1, showing that in this case there are more countries with environmental efficiency that could be improved. Countries with improved environmental efficiency are also found on the periphery, like Portugal, or even in the north, like Lithuania.

Thus, using method 1, it can be concluded that out of the 27 countries studied in the given period of time, 13 countries have excellent environmental efficiency compared to the others, another 2 have good efficiency, 6 of them have an average value and another 6 can improve.

As can be seen, countries like Germany, Italy, or France have excellent environmental efficiency compared to Greece, Romania, or Hungary, whose environmental efficiency is better compared to the others.

Now, when the method is changed, it can be seen that of the 27 countries, only 9 have excellent eco-efficiency, while with method 1 there were 13. Two of them have good eco-efficiency as before, 6 remain average as before, but now we have 10 countries within the category of poor or improvable eco-efficiency.

Commenting on the most significant results of both methods, we have:

- Malta, Sweden, the Netherlands, Germany, Denmark, Austria, Italy, France and Luxembourg maintain excellent eco-efficiency. Belgium remains within a good eco-efficiency. Spain, Finland and Cyprus continue to maintain a medium eco-efficiency, and Greece, Romania, Slovakia, Hungary, the Czech Republic and Bulgaria continue with eco-efficiency that could be improved.
- Latvia goes from having excellent eco-efficiency to medium eco-efficiency.
- Lithuania goes from having an excellent eco-efficiency to one that could be improved.
- Ireland goes from excellent to good eco-efficiency.
- Poland goes from excellent to average eco-efficiency.
- Estonia goes from having good to a medium eco-efficiency.
-Croatia, Slovenia and Portugal go from having an average eco-efficiency to one that could be improved.

In conclusion, the number of countries with excellent and good eco-efficiency has decreased when passing from method 1 to method 2, while those with medium eco-efficiency have remained. The number of countries with eco-efficiency that could be improved has also increased.

The first observation that draws attention is that the values obtained by method 2 are lower than those obtained by method 1. This is mainly because more weight has been given to methane emissions, causing this unwanted output to cause a decrease in the eco-efficiency of the country and making method 2 more precise since it delves a little deeper into the variables used to perform the calculations. This is so because, thanks to considering different weights in, for example, GHGs, method 2 allows us to find peculiarities that method 1 does not consider. A clear example is the case of Lithuania, which will serve us later to introduce the MAM to delve deeper into the results obtained.

### Analysis of environmental efficiency for each of the countries of the European Union

In order to try to explain the environmental efficiency results obtained, we wanted to comment on what happened in each country throughout the period studied.

Germany, which has excellent eco-efficiency, has very high economic indicators (higher GDP in the EU) that help to offset the fact that it is one of the countries that emit the most GHG emissions. If it were not for its economic indicators, we would be talking about a country with low eco-efficiency. The main solution is to reduce these emissions by reducing the use of coal (Germany is the first country in the EU that imports the most coal) or using more electric vehicles.

Austria also presents excellent eco-efficiency for both methods thanks to the fact that it has a high GDP and not so high GHG emissions thanks to its energy policy with its strong commitment to hydroelectric power.

Belgium also has a high eco-efficiency but does not maintain excellence (see “Appendix, Fig. 12”) since, although its economic indicators are high (similar to those of Austria), it has yet to reduce its GHG emissions.

Bulgaria is one of the countries with improvable eco-efficiency that still uses a lot of coal as a reserve source to produce electricity. Being one of the poorest countries in terms of GDP does not help improve these values.

The case of Cyprus is peculiar because it presents an excellent eco-efficiency in the first years (see “Appendix, Fig. 13”). Then it does not improve, being located within the medium category. Its economic indicators are generally low compared to other countries. Its emissions could fall further as Cyprus remains heavily dependent on oil as an energy source (by 2020, oil-based electricity generation will exceed 85%).

Croatia goes from having an average environmental efficiency to one that can be improved by changing the method. In 2017, it presented excellent eco-efficiency as a reward for its slow economic growth (see “Appendix, Fig. 13”). Its GDP, for example, is higher than that of Cyprus, but it still has to cut its emissions a lot to increase eco-efficiency. It must still improve its energy policy to bet on renewable sources and improve its economic parameters to increase eco-efficiency.

Denmark is one of the countries with excellent eco-efficiency for both methods. It has been reducing its reliance on coal and oil throughout the period while investing in renewable sources. Its economic indicators are also high and help it achieve excellent eco-efficiency.

Slovakia has low eco-efficiency mainly due to its low economic indicators, although it has been reducing its dependence on coal and oil while continuing to invest in nuclear power.

The case of Slovenia also makes it have a low eco-efficiency (medium to improvable by changing the method) due to its low economic indicators. It still depends on sources such as coal while using nuclear power.

Spain is a country that presents medium environmental efficiency. Despite having been betting on renewable energies, it continues to have high emissions, which, accompanied by not very high economic indicators, end up harming it and placing it in a category that could be better than what is expected.

According to the method, Estonia, which is between good and medium eco-efficiency, has been phasing out coal as a source of electricity, just like oil. For most of the period, method 1 maintains excellence (see “Appendix, Fig. 12”), although it is hampered by slow economic growth. Nuclear power has been non-existent in the country. Estonia is penalized from the point of view of environmental efficiency by economic parameters since GDP and GDP per capita are low compared to other countries.

Finland is in the middle category, according to the data used in this project. Finland is a country whose electricity does not come mainly from coal and oil, further decreasing over time. Nuclear power is still present at an average of 33%, while other energy sources are used. The low values of the GDP accompanied by emissions around the average mean that it has an average value of environmental efficiency when it could be excellent if the GDP increased with respect to its current value.

France is one of the countries that has invested the most in energy due to that of nuclear origin since it provides an average of 70% of the country's electricity while coal and oil continue to lose weight. It is one of the countries with excellent environmental eco-efficiency since it has the second highest GDP in the European Union and shows a downward trend in GHG emissions. However, it has the highest values in the European Union regarding methane emissions because although transport is the most polluting sector, agriculture occupies the second place. In order to continue increasing these efficiency values or for France to maintain them, it would be necessary to progressively reduce these emissions while maintaining or improving the country's GDP.

Greece is one of those countries whose environmental efficiency is very close to the average but ends up entering the eco-efficiency category that could improve by a few tenths. The country still uses energy sources such as coal and oil to produce electricity, but with a downward trend over the years of coal use. Polluting emissions in Greece are higher than those produced, for example, in Denmark. This, together with having one of the lowest GDPs in the European Union, causes its eco-efficiency to drop too low.

Environmental efficiency in Hungary is also at levels that could be improved as they are quite low (below 30%). Electricity production from coal and oil sources shows a downward trend in the country. Hungary continues to bet on nuclear energy since its use to produce electricity in the period studied is around 45–50%. Despite this, Hungary has the disadvantage of having a lower GDP per capita and GDP than countries like Greece.

In Ireland, there are very good environmental efficiency values, while method 1 is considered excellent. allows to obtain a good eco-efficiency because the weight of methane emissions reduces it. It is especially in 2012 (see “Appendix, Fig. 12”) also due to the financial crisis that the country was going through. Ireland has a strong economy, standing out for finishing the study period as the second country with the highest GDP per capita (industrial production index increased in 2020), while most countries suffered a drop in growth. The fact that the environmental efficiency drops to good in method 2 is due to the high methane emissions and, if reduced, it would have more than excellent eco-efficiency. Policies to reduce such emissions have already been implemented in the livestock sector (Teagasc 2021).

Italy, with excellent eco-efficiency, has spent a decade reducing the use of oil and coal for electricity while promoting other energies without ever using nuclear energy. Italy has a relatively high GDP offset by high polluting emissions.

Latvia has excellent eco-efficiency according to method 1, while according to method 2, it is in the category of medium eco-efficiency. Latvia's GDP is low, but having lower polluting emissions makes it have high environmental efficiency values. By giving more weight to methane emissions, they lower the country's environmental efficiency to the average level, but without going below 70%.

Lithuania is a strange case because you go from having excellent environmental eco-efficiency to improved environmental efficiency when we change the method and give more weight to methane emissions. Emissions are lower than in other countries, but when methane is considered with more weight, it means that not being a country with a very high GDP per capita and GDP, the efficiency drops. Lithuania can solve this problem mainly by reducing polluting methane emissions and improving the economy to counteract the problem.

Luxembourg has excellent eco-efficiency for both methods since its energy does not come from sources such as coal or oil but will originate from renewable sources that result in not too high emissions. Furthermore, its GDP and high GDP per capita make it an excellent country from an environmental point of view.

Malta has excellent environmental efficiency by both methods, as it has a growing GDP per capita, even higher than countries like Hungary or Latvia. To this, we must add that although Malta started at the beginning of the study period as a country whose electricity came mainly from oil, it had been reducing it to almost 3% in 2020. This translates into lower greenhouse gas emissions with the passage of time and, therefore, an improvement in environmental efficiency.

The Netherlands has high economic indicators that, accompanied by electricity production that ceases to depend over time on energy sources such as coal or oil (which translates into a reduction in GHG emissions), allow the country to have excellent environmental efficiency compared to other countries of the European Union.

Poland has an average environmental efficiency for method 2, while method 1 is considered excellent (Fig. 11). Poland still gets part of its electricity production from sources like coal (mainly) and oil. Although its trend has been decreasing over time, it can be seen that CO2 and CH4 emissions are high. These emissions are offset by a high GDP, which means that eco-efficiency can be improved, since the moment more weight is given to methane emissions, the country's eco-efficiency suffers a decrease.

Portugal has an environmental efficiency between medium and improvable by both methods, marking a constant trend by method 1 with exceptions in 2012, 2019 and 2020 (see “Appendix, Fig. 13”). Portugal has been reducing its electricity production from coal and oil throughout the years. Despite reducing these energy sources, emissions did not experience a very pronounced decrease because Portugal has a very large vehicle fleet that contributes to them. The fact that the country's economic indicators (GDP and GDP per capita) are not too high helps ensure that the drop in efficiency continues.

The Czech Republic has relatively low environmental efficiency with both methods. The country continues to use coal to produce electricity despite being reduced over the years while it is committed to nuclear energy since it reached 36% of production in 2020. GDP and GDP per capita are not valued very high, slightly below the European Union average. Additionally, GHG emissions are high and affect environmental efficiency, so they continue to decline. The perfect solution for this country is to continue reducing GHG emissions while the economy continues to improve.

Romania presents a similar case to the Czech Republic since its environmental efficiency is relatively low; that is, it could improve. It is a country that still depends on coal to produce electricity, but that shows a downward trend. In this case, CO2 emissions are lower than in the Czech Republic, but CH4 emissions are higher. Regarding the economic indicators, both GDP and GDP per capita are not very high in this case and cause environmental eco-efficiency to drop to very small values numerically.

Sweden has excellent environmental eco-efficiency. It is a country that has coal for the production of electricity and oil but still maintains nuclear energy for the production of electricity. It has a high GDP per capita and a GDP within the average, which, together with low GHG emissions, allows it to have high-efficiency values.

- For the countries that remain within the category of improvable environmental efficiency, some peculiarities can be commented on by observing Fig. 14 of the Appendix
- 2014: Countries with a downward trend show excellent eco-efficiency, such as Greece, Lithuania and Slovenia for method 2, mainly due to decreased emissions.
- 2019 and 2020: Minimum emissions are marked (in 2020, especially due to the COVID pandemic), as is the case with Romania, which also experiences an increase in economic indicators.
- 2012 and 2017: Portugal and Croatia set maximums these years.

### Unification of methods: average environmental efficiency of each of the countries and the whole of the EU-27

Once both methods have obtained the results, it is necessary to unify both to have a definitive classification of the 27 countries of the European Union in terms of environmental efficiency (Table 5).

As can be seen, those with excellent environmental efficiency are Malta, Denmark, Germany, Italy, Austria, the Netherlands, Sweden, France and Luxembourg. Ireland, Belgium and Latvia have slightly lower values and are in the good eco-efficiency category.

Poland, Finland, Spain, Estonia, Lithuania, Cyprus and Portugal occupy the average values. In contrast, the lowest values and whose eco-efficiency should be improved are Slovenia, Croatia, Greece, Romania, Slovakia, Hungary, the Czech Republic and Bulgaria.

After having carried out the calculations and drawn the first conclusions for each of the countries of the European Union, it has been considered necessary to place the category in which the European Union is as a whole.

Basically, the average of all the countries has been obtained by each of the two methods to obtain the average in each of the years and, of course, the total average of the European Union in those eight years.

As seen in Table 6, the evolution of the values of method 1 shows that it has practically remained close to the category classified as good but consolidating in the average. As for method 2, there are no doubts, and it is clear that the tendency is to stay in the category of medium environmental efficiency.

Observing the data, it is the year 2014 in which we find excellent eco-efficiency in the most significant number of countries. This is explained by analyzing the context of the European Union:

- Industrial production index: increased by almost 2% after going through several years of decline due to the economic crisis that hit a large part of the continent's countries.
- The **economic indicators** (GDP and GDP per capita) show growth of 2.3% and 2.19% with respect to 2013, which began to be a sign that the continent's economic recovery was beginning to be a reality.
- Finally, concerning **GHG emissions** (methane and carbon dioxide), there was a decrease of up to almost 5% compared to 2013 for CO2 and 1.5% for methane.

In 2020, by contrast, it was the year with the fewest countries in the excellent eco-efficiency category, as the industrial production index fell by as much as 5% and economic indicators fell by 4.5%. Although there are indeed some countries that, thanks to the drop in GHG emissions, experienced a small increase in their eco-efficiency (not very abrupt), such as Bulgaria, Cyprus, Spain, Lithuania and Poland. The case that most attract attention is that of Cyprus, since its economic growth, although slow in previous years, accompanied by the decrease in its emissions, has allowed it to achieve better eco-efficiency in 2020 compared to other EU countries. 27. In the end, this happens because eco-efficiency not only takes into account emissions but also takes into account economic indicators, and the eco-efficiency of a country depends on the situation of others since it is also measuring environmental efficiency relative, as we have said before (Table 8).

**Table 8:**
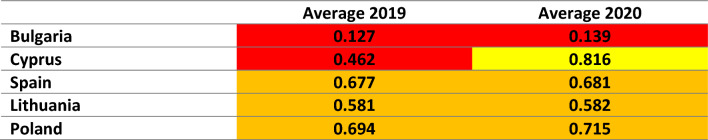
Comparison of the change in eco-efficiency from 2019 to 2020 in the countries that have increased eco-efficiency

### Comparison between the period 2005–2012 and 2012–2020

The idea of this section is to compare this study and the one carried out in the article “Measuring environmental efficiency in the countries of the European Union with the improved method of environmental data analysis (DEA) during the period 2005–2012. (Hermoso-Orzáez et al. 2020) to find out how eco-efficiency has changed in both periods and have a broader understanding of what has happened in these 15 years. Note that the main caveat is that the UK was included in the above study as it was part of the European Union at the time and would therefore have influenced the overall eco-efficiency of the European Union.

**Fig. 3:**
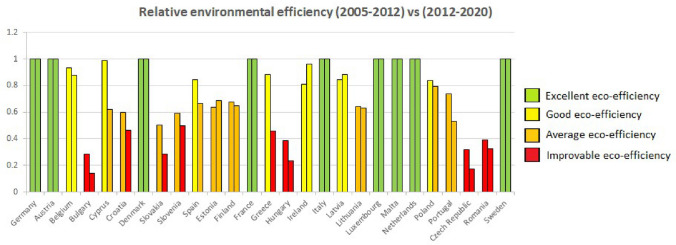
Graphic comparison of the 27 countries of the European Union for the two periods between 2005–2012 and 2012–2020

The first conclusion (Fig. 3) that can be seen between both periods is that more countries have experienced a decrease in their eco-efficiency than those that have experienced an increase. There are also, of course, countries where it has been maintained.

It is curious to note that no countries have achieved excellent relative environmental efficiency over the years. However, those that have already done so, such as Austria, Denmark, France, Germany, Italy, Luxembourg, Malta, the Netherlands and Sweden, continue to maintain it in the new period studied.

On the other hand, some countries have increased their eco-efficiency, such as Estonia, Finland, Ireland and Latvia. The most striking case is that of Ireland since it was on the border between good and average eco-efficiency and has positioned itself close to excellence due to its continuous improvement over the years in terms of GDP and emissions.

The study results show that several countries have a reasonably high eco-efficiency, which places them in the excellent category, such as Germany, Austria, Denmark, France, Italy, Luxembourg, Malta, the Netherlands and Sweden.

These countries already had excellent eco-efficiency in the period between 2005 and 2012. Among these countries are the founding countries of the European Union (Table 9), except Belgium, which, although it has high eco-efficiency, is not within excellence, having also experienced a small reduction with respect to its eco-efficiency in the study of the period between 2005 and 2012.

**Table 9:** Summary table with the year of entry of each of the countries of the European Union and its eco-efficiency in the period between 2012 and 2020

Year of income	Countries	Excellent eco-efficiency	Good eco-efficiency	Average eco-efficiency	Improvable eco-efficiency
1957 (founders)	Germany, Belgium, France, Italy, Luxembourg and the Netherlands	Germany, France, Italy, Luxembourg and the Netherlands	Belgium		
1973	Ireland and Denmark	Denmark	Ireland		
1981	Greece				Greece
1985	Spain and Portugal			Spain and Portugal	
1995	Austria, Finland and Sweden	Austria and Sweden		Finland	
2004	Estonia, Latvia, Lithuania, Poland, Czech Republic, Hungary, Slovakia, Slovenia, Cyprus and Malta	Malta	Estonia, Latvia	Lithuania, Poland and Cyprus	Czech Republic, Hungary, Slovakia, Slovenia
2007	Bulgaria and Romania				Bulgaria and Romania
2013	Croatia				Croatia

It must be taken into account that the UK has no longer been present in this study because it left the European Union in 2020 and, therefore, has affected the average of the group of countries by having excellent eco-efficiency in the first period (2005–2012).

What the countries with the best eco-efficiency have in common are those that make up the Central European zone since the more we are located in Eastern Europe, we find countries with low environmental eco-efficiency, such as Hungary, Bulgaria, Romania, Greece, Croatia, the Czech Republic, Slovenia and Slovakia.

On the other hand, many countries have a medium or low environmental eco-efficiency compared to those that are excellent, and, highlighting those that entered in 2004, only Malta has excellent eco-efficiency, one of the countries with the greatest dependence on oil. In 2016 Malta decided to change its energy policy so as not to depend so much on oil to produce electricity. Latvia is another of those countries where environmental and energy policies are working as it has a good eco-efficiency compared to countries that entered in 2004, such as Slovakia, Slovenia, Estonia, Hungary or Lithuania.

Lithuania is an exceptional case as it presents excellent eco-efficiency if we consider the same weight for both CO_2_ and CH_4_ emissions. However, when we decide to give more weight to methane, this eco-efficiency drops to values of 0.25, which, when making the average between both methods, places the country in a category of medium environmental efficiency.

The last countries to join the European Union were Romania and Bulgaria in 2007 and Croatia in 2013. These three countries have an eco-efficiency that could be improved, with Bulgaria having the lowest value in the entire European Union (0.1423).

Among the rest of the countries that experience a decrease in their efficiency values, we find some quite curious cases, such as Cyprus, since it goes from having almost excellent environmental efficiency values to values located within the average.

**Fig. 4:**
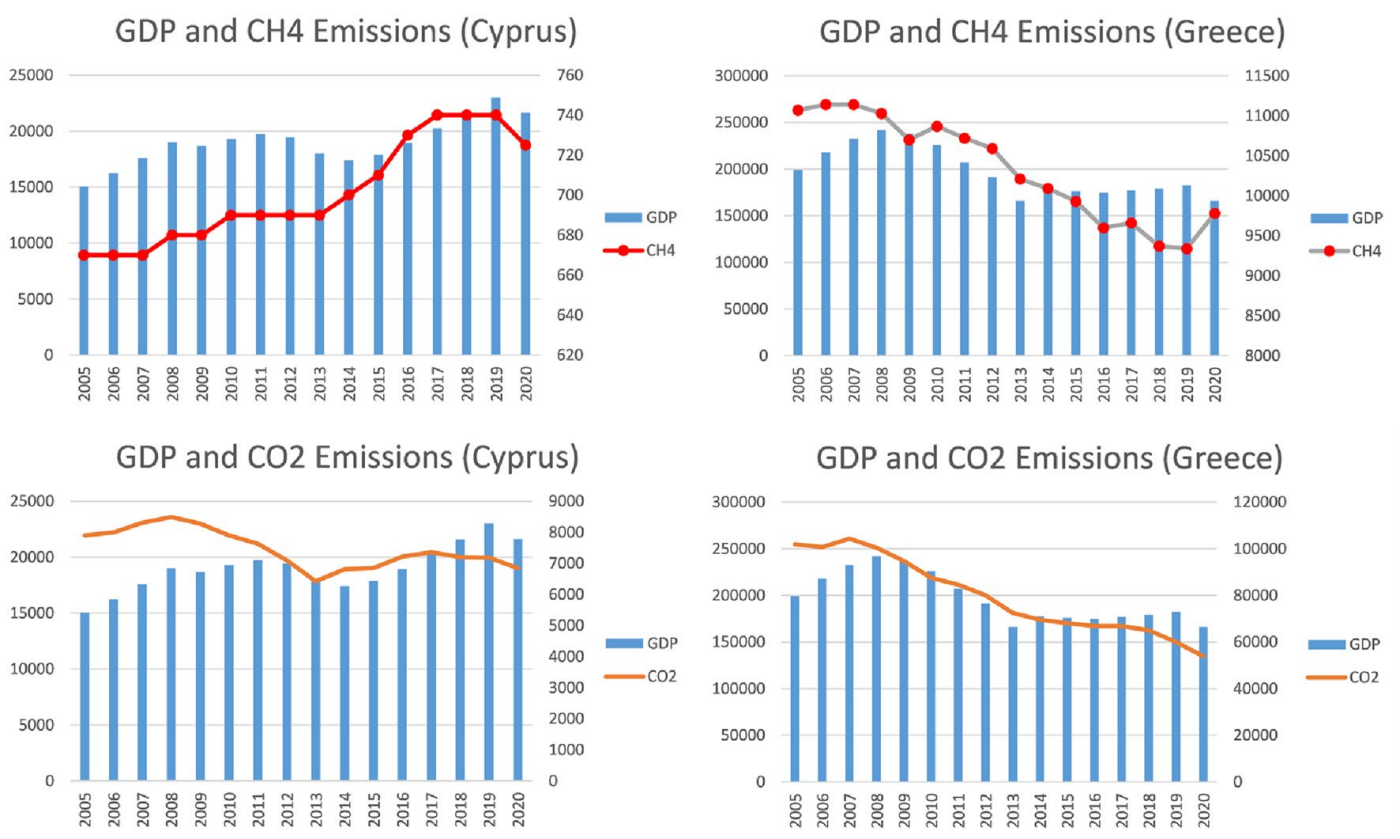
Relationship between GDP and GHG emissions for the countries of Cyprus and Greece from 2005 to 2020

To put in context what could have happened in Cyprus (Fig. 4), the GDP and emissions data have been reviewed since they are the main values determining efficiency.

In Cyprus, they have been affected in terms of eco-efficiency because methane emissions have presented a line that has been progressively increasing while CO_2_ decreased but was still high.

We must also look at the case of Greece (Fig. 4) since it presented a good eco-efficiency in the first period. All its emissions have decreased over time, but its economic indicators have experienced a decline due to the economic crisis that the country experienced since the end of 2009 or the crisis it experienced in 2012 (Katsikas 2014). This has undoubtedly harmed it in terms of eco-efficiency.

Finally, in the case of Spain, it has gone from a good eco-efficiency to another located in the average since the country's GDP, despite being in fourth place among the countries of the European Union, began to experience a decrease from 2008 due to the economic crisis and, not reaching values prior to this year until almost a decade later.

**Fig. 5:**
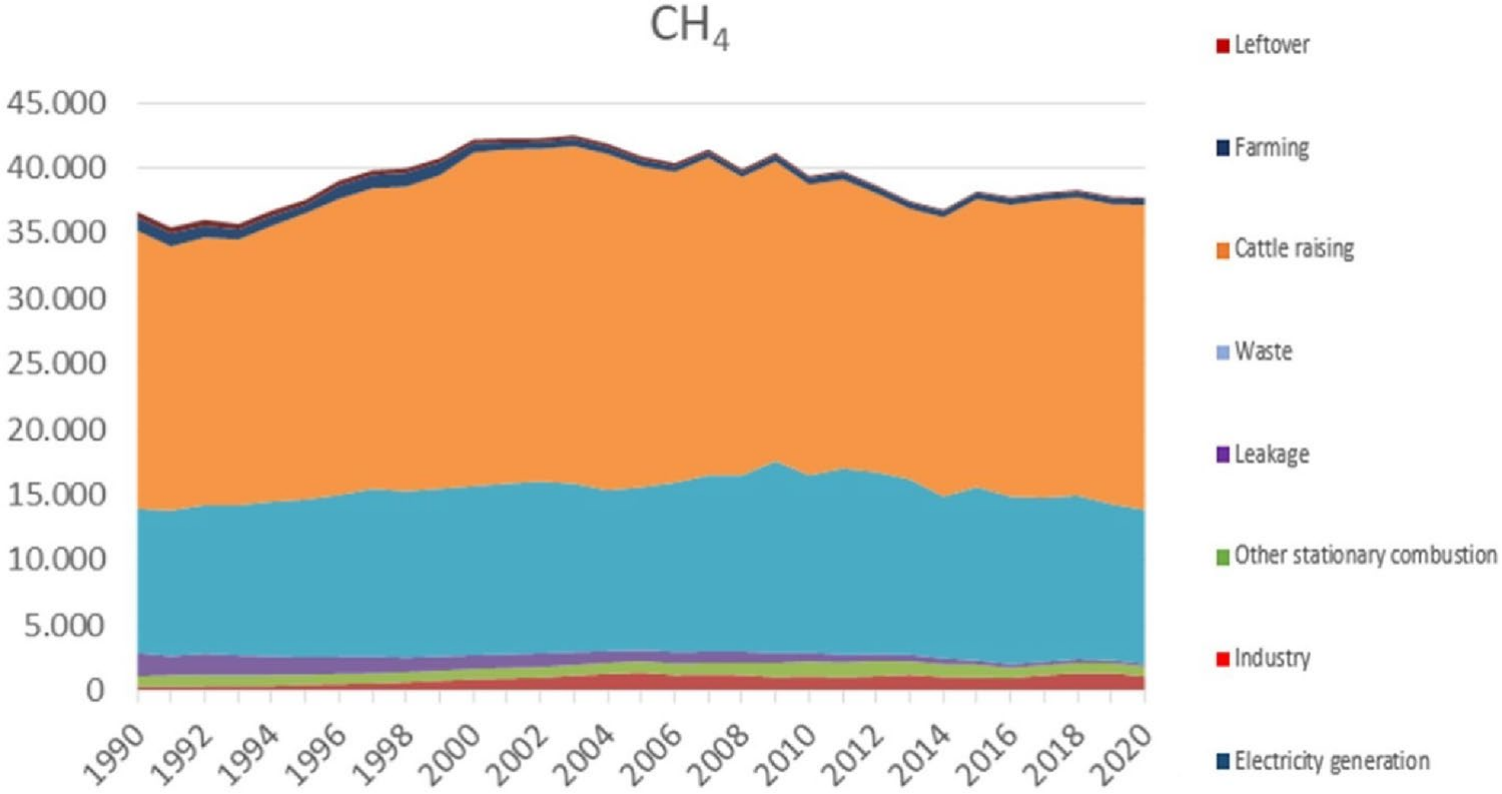
Gross emission of CH _4_ (kt CO _2_ -eq) by aggregated activities in Spain (Ministerio para la Transición Ecológica y el Reto Demográfico 2022)

However, Spain has not done everything wrong so that eco-efficiency values have dropped in this way in recent years, so it is necessary to comment on what has happened in both parameters related to eco-efficiency:

- Economic indicators: Since 2005, the GDP growth in Spain has not been very positive. This growth is closely related to the economic crisis that broke out in 2008 (end of the real estate bubble, banking crisis, increase in unemployment, etc.) (Daher 2013) and which, according to the INE (Spanish National Statistics Institute), ended in 2014. Although the INE said, it concluded that year, it was not until 2016 that values similar to those of 2008 were reached. In 2020 the GDP would hit another drop due to the pandemic caused by COVID-19.
- **GHG emissions:** Carbon dioxide emissions have decreased since 2005, while methane emissions, despite experiencing a decrease, have also begun to increase since 2014.
- *CO*_*2*_* emissions*_*:*_ The fact that carbon dioxide emissions are experiencing a decrease shows that Spain's commitment to renewable energies is working through the decarbonization plan initiated in 2007 (RevistaHaz 2022).
- *CH*_*4*_* emissions*: These emissions have been a small increase in this last period. The sectors that contribute the most to these emissions in Spain are the livestock and waste sectors (Fig. 5).

### Enhanced Analysis (MAM)

To conclude the study carried out, the values obtained serve to have an idea of what affects eco-efficiency, but, indeed, it is not possible to differentiate how some indicators affect it, and it happens that cases such as Lithuania appear. 

**Fig. 6:**
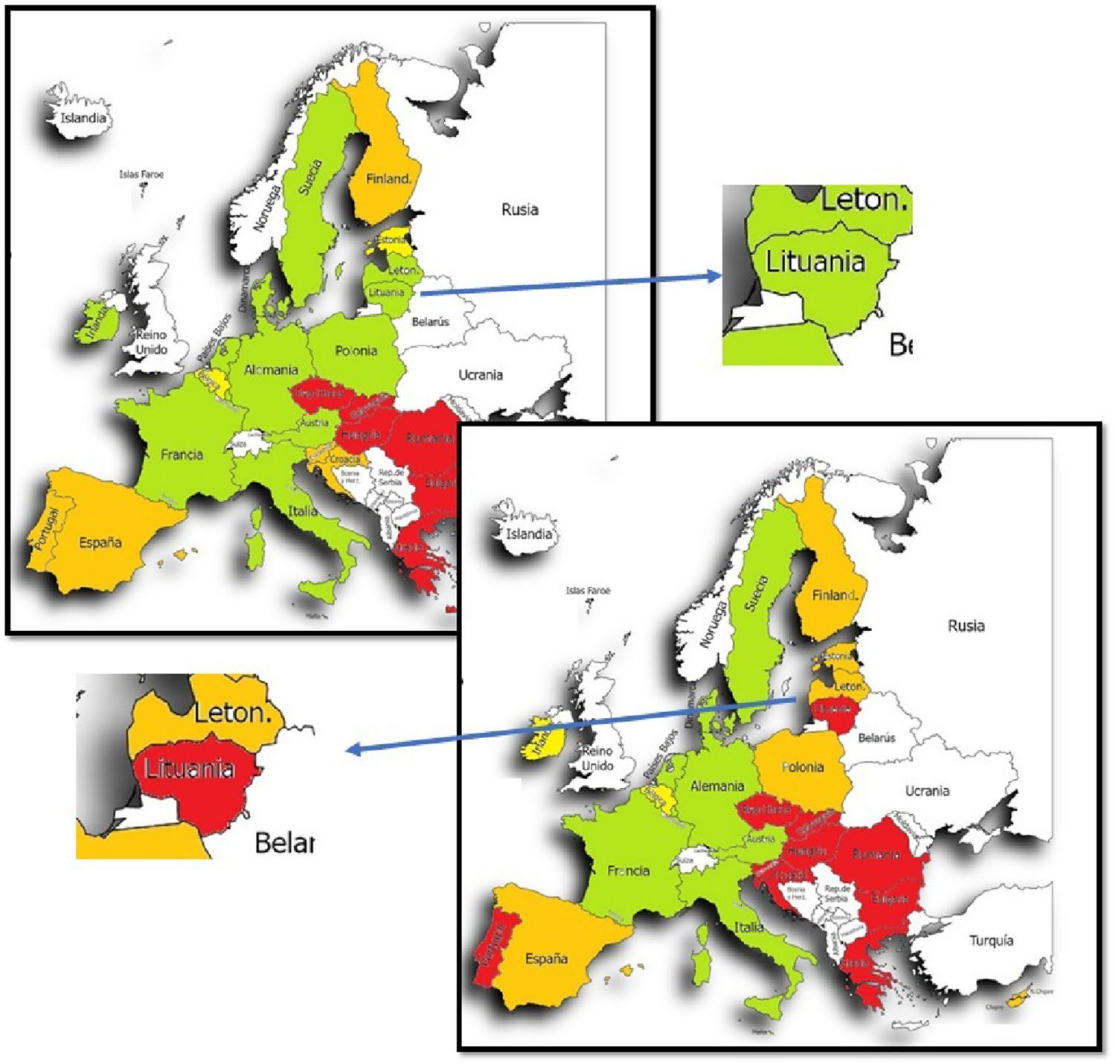
Situation of environmental efficiency for the case of Lithuania differing between both methods

Figure 6 goes from having an excellent eco-efficiency by method 1 to having an eco-efficiency that could be improved by method 2.

Because of the results obtained, an attempt has been made to explain it by studying the evolution of the available data. However, it is not possible since methane emissions decrease simultaneously as the economic indicators increase throughout this period.

As a result, it is necessary to analyze the results in another way that allows biasing the values to save the disparate differences between some countries, for which it is necessary to use the improved analysis method (MAM).

**Fig. 7:**
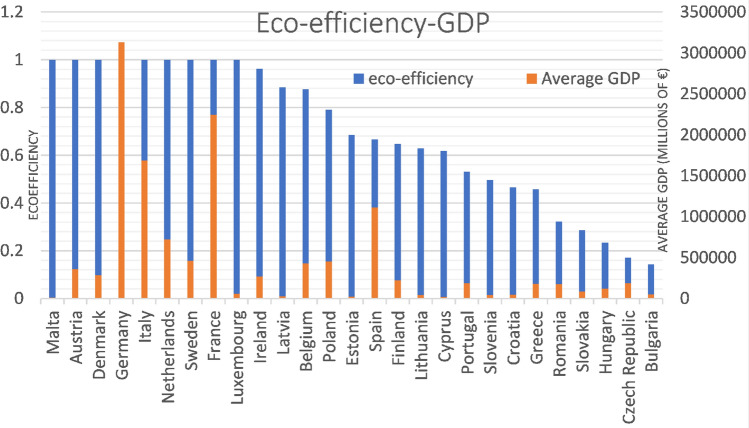
Comparison between environmental efficiency and average GDP for the 27 countries of the European Union in the period between 2012 and 2020

First, the comparison will be made with the average environmental efficiency obtained by both methods in the period studied and for each of the countries of the European Union with the average GDP for each of these countries (Fig. 7). It can be seen that some countries have an eco-efficiency very similar to 1, which is the highest value, while their average GDP differs, as is the case of Malta, Denmark and Germany, to give a few examples. In addition, it can be seen that Germany has the highest GDP in the entire European Union. In contrast, Spain has the fourth highest GDP and an eco-efficiency around the average, placing it in the fifteenth position.

These comparisons are intended to establish a hierarchical order as precisely as possible, exposing other indicators such as GDP per capita and unwanted outputs related to methane and carbon dioxide emissions.

**Fig. 8:**
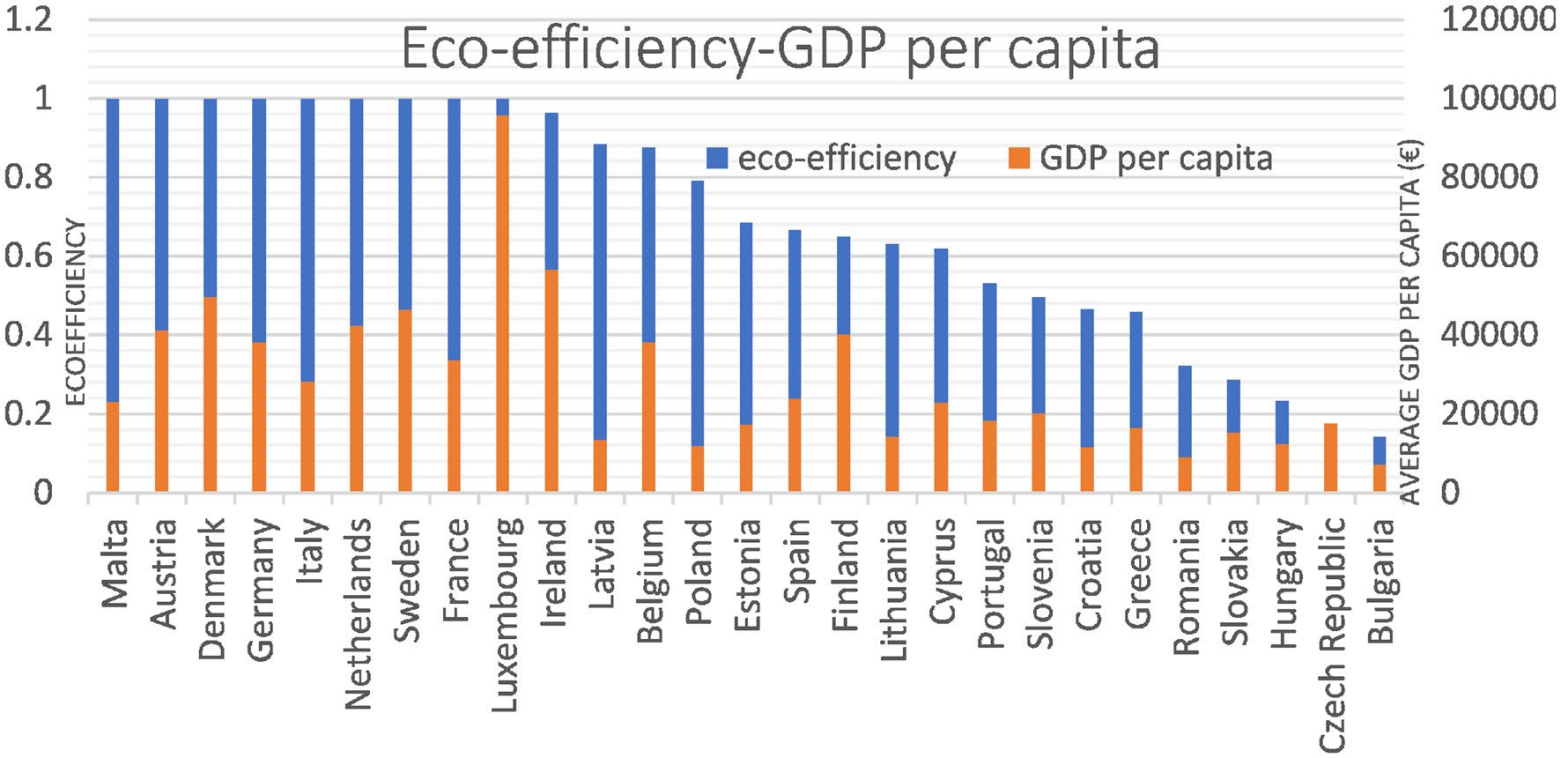
Comparison between environmental efficiency and average GDP per capita for the 27 countries of the European Union in the period between 2012 and 2020

Looking at GDP per capita (Fig. 8), Luxembourg is the one with the highest value and also has excellent eco-efficiency. On the other hand, the second country with the second highest GDP per capita ends up occupying a tenth place in terms of eco-efficiency values.

The case of Cyprus and Malta is curious since both have quite similar incomes. However, although Malta is the most eco-efficient country, it turns out that Cyprus has an average eco-efficiency that places it in 18th place.

**Fig. 9:**
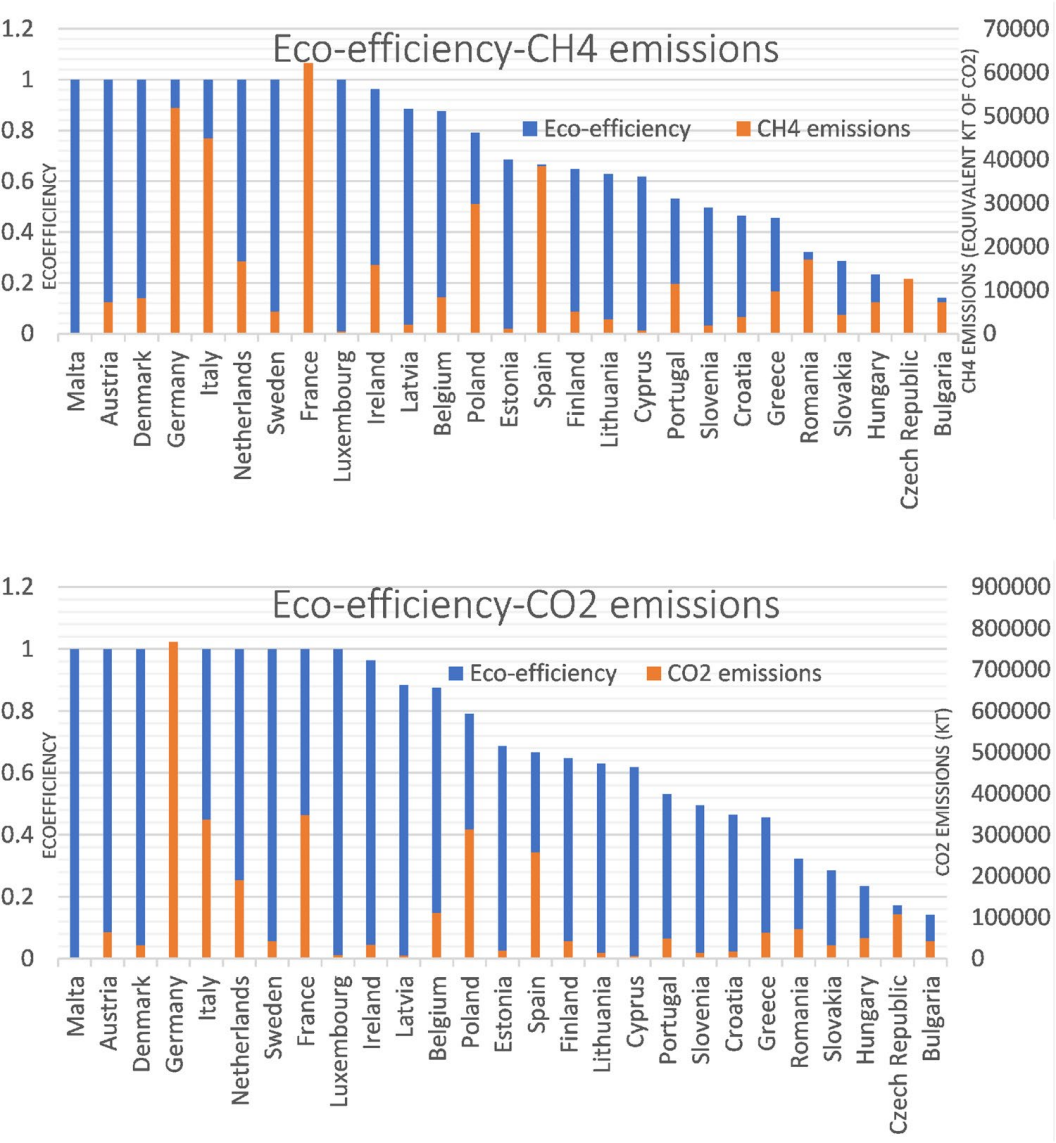
Comparison between environmental efficiency and GHG emissions for the 27 countries of the European Union in the period between 2012 and 2020

Observing the figures that compare eco-efficiency with GHG (Fig. 9), it can be seen that there are countries like Germany and France that are the ones that emit the most GHG, and even so, their eco-efficiency is not affected. This is because they are also the countries with the highest GDP in the European Union, allowing us to highlight a positive relationship between them. After all, they are acting more efficiently than all neighboring countries.

As for Spain, it can be seen that it is the fourth country with the most methane emissions and the 5th with the most carbon dioxide emissions, which, in terms of GDP, also ranks fourth. This dramatically affects its eco-efficiency since it places it around the average of all the countries of the European Union. This has also been compared with the first study period, where it was the sixth country with the most methane emissions, and at that time, its eco-efficiency was better.

As can be seen, countries with relatively low eco-efficiency share few emissions as they are not acting as efficiently as possible. Suppose the graph that compares eco-efficiency with methane emissions is carefully observed, and we look specifically at the case of Lithuania. In that case, it is explained that this amount of methane, compared to other countries, having more weight than CO2 emissions, affects its eco-efficiency. In addition, your GDP also ends up affecting you negatively. If we look at the graph comparing eco-efficiency to GDP, it can be seen that Lithuania has a very low value compared to other European Union countries.

**Fig. 10:**
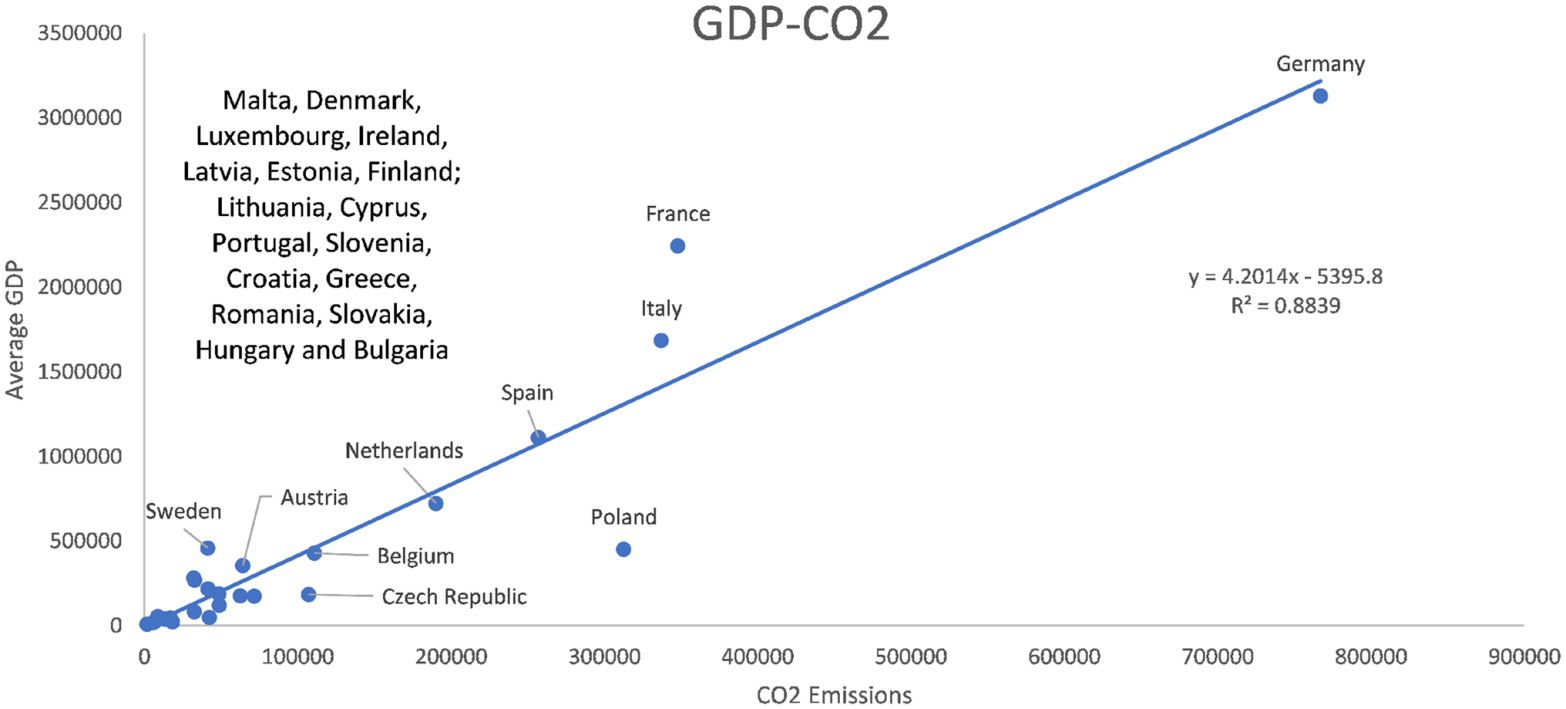
Ratio between the average values of GDP and the average values of CO2 emissions for the 27 EU countries in the period 2012–2020

**Fig. 11:**
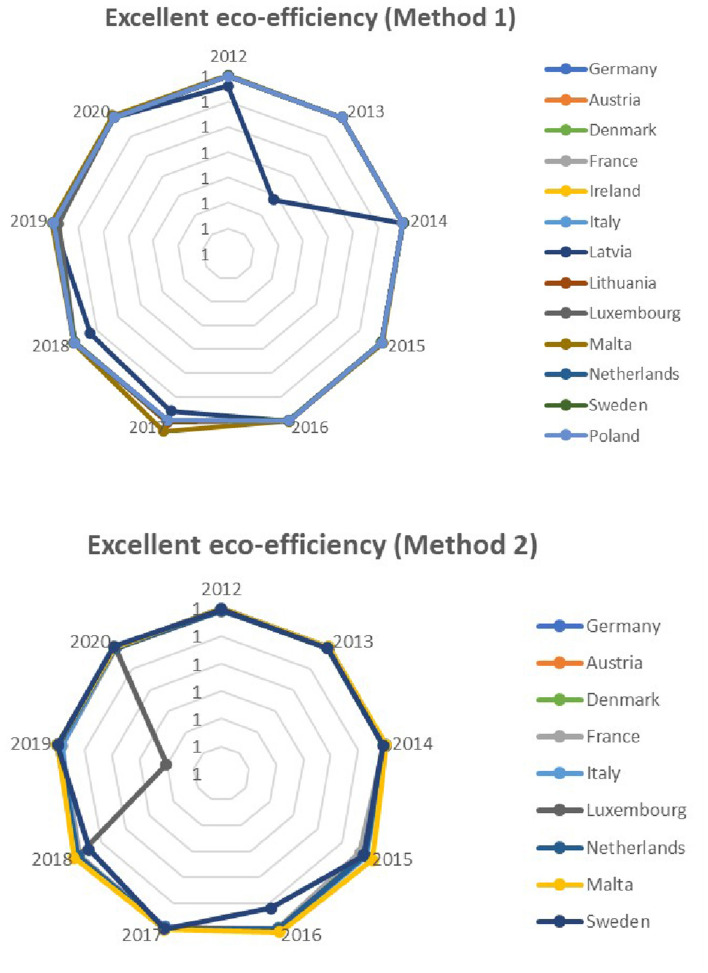
Temporary variation of eco-efficiency for those countries of the European Union within the excellent category

**Fig. 12:**
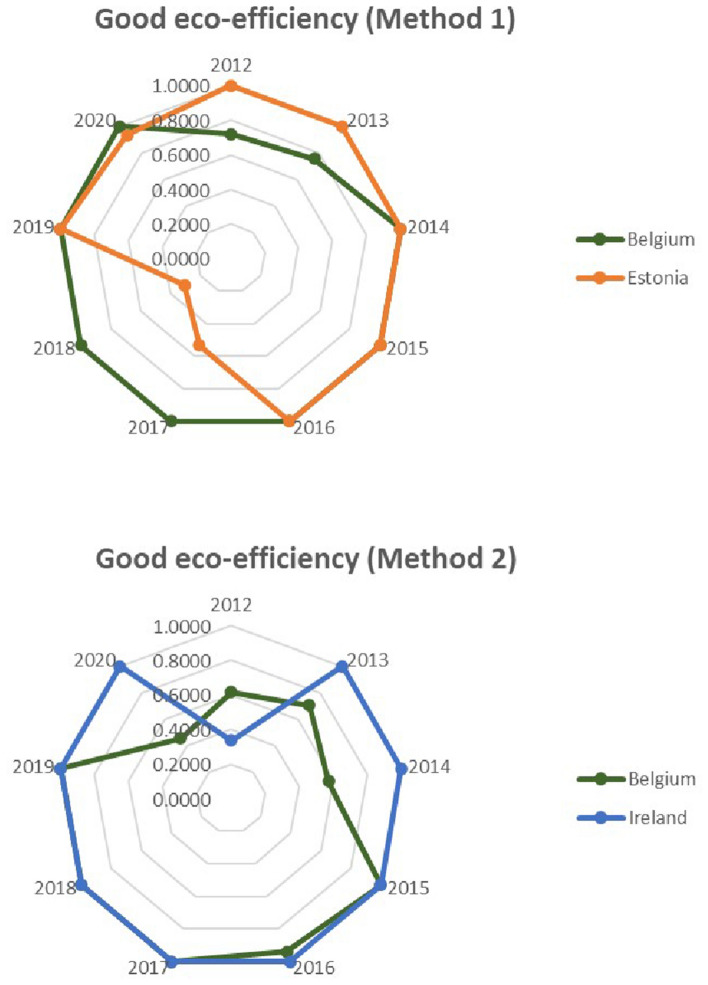
Temporal variation of eco-efficiency for those countries of the European Union within the good category

**Fig. 13:**
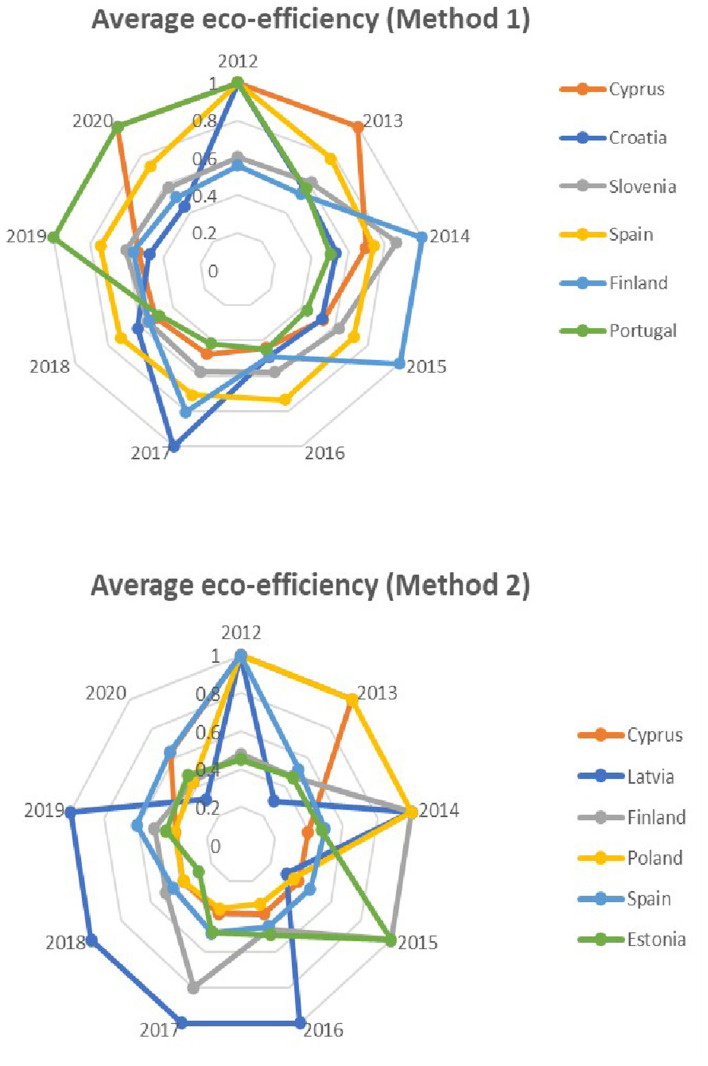
Temporary variation of eco-efficiency for those countries of the European Union within the average category

**Fig. 14:**
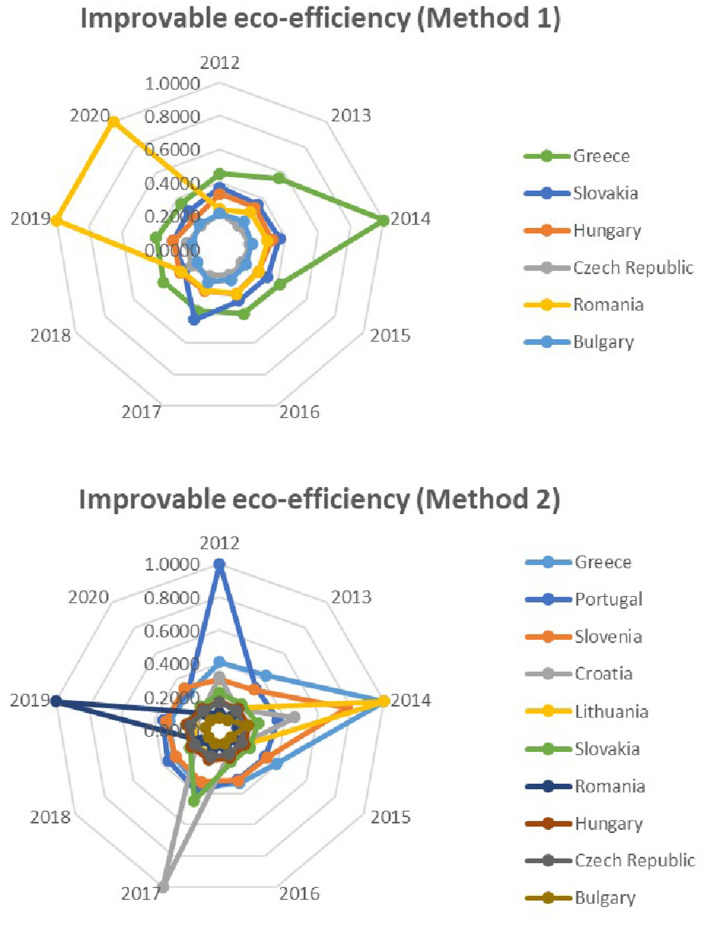
Temporary variation of eco-efficiency for those countries of the European Union within the improvable category

As shown in Fig. 10, the relationship between GDP and CO2 emissions is positive because the countries with the highest GDP are also those with the highest carbon dioxide emissions, with Germany, France, and Italy being the countries with higher GDPs. However, Spain, the Netherlands and Poland are in a reasonably intermediate zone, while all the others are already in the low zone.

Of the intermediate zone, it is curious that the Netherlands is already the most efficient, despite being relatively close to Spain. This may be due to its higher emissions, which harm the value of its eco-efficiency. In addition, there is the case of countries that, although their emissions are low, show that they do not present an optimal situation because they are countries that are not as industrialized and, therefore, their eco-efficiency cannot be as good as that of other countries in the European Union.

To finish this study, it is necessary to comment, given what has been obtained, on a set of solutions (although previously it has been dropped in a particular way) that will be necessary to apply to improve the countries' environmental efficiency. These could be:

- Achieve greater energy independence that allows the different countries to be more self-sufficient: This is related to the dependence of some countries on various energy sources whose supply depends on other countries, such as coal, oil, or natural gas. Promoting an economy based on renewable energies with the support of clean sources of emissions such as nuclear energy would encourage growth from the point of view of environmental efficiency since, in addition, the economic cost would be lower if third countries do not set the price. Within this, we find the case of Spain since it is a country with very optimal conditions to promote renewable energies that could be supported by nuclear energy because it has one of the most important uranium reserves in the European Union. In addition, there are still some countries that need a review of their energy policy such as Bulgaria, Croatia, Poland, the Czech Republic, Cyprus, Greece, Romania, Malta and Slovenia. Germany can also improve its energy policy as it continues to use a large amount of coal today, becoming the country, according to Eurostat, that imports the most coal from the entire European Union (32% of the total) and is also the country that imports the most natural gas together with Italy, the Netherlands and France.
- Reduce GHG emissions: One of the keys to increasing the eco-efficiency of a country, as shown in this study, is the reduction of greenhouse gas emissions. Compared to the first study, CO2 emissions show a downward trend, reducing by almost 8.25% between this study period and the previous one. Methane emissions also show a downward trend, presenting a global reduction of 8% between one period and another. However, although there has been a reduction in both greenhouse gases, some countries such as Germany, Poland, Italy, France and Spain still have high emissions. Germany or France are countries that hold their own in terms of eco-efficiency because they cushion these emissions with a reasonably high GDP. However, when it comes to Spain, it can be seen that these emissions, accompanied by the fact that it is not a highly industrialized country, still weigh it down in terms of eco-efficiency. Poland is undoubtedly one of the countries most affected from the point of view of emissions since methane emissions are given greater weight than carbon dioxide emissions. Its eco-efficiency drops to the average value of 0.5648 when it could have a good eco-efficiency very close to excellence because it also has one of the highest GDPs in the European Union. The countries that, without a doubt, should rethink their environmental policy are Bulgaria, Cyprus, Croatia, Slovakia, Slovenia, Estonia, Greece, Hungary, Lithuania, Poland, the Czech Republic and Romania. Ireland also has high methane emissions and has already implemented plans to reduce emissions in the livestock-related sector.
- Rethinking the economic model in order to create a more sustainable one that at the same time increases GDP and GDP per capita: Thanks to this study, it has been possible to observe that within the European Union, there are countries that, despite having a decrease in their greenhouse gas emissions, fail to achieve good environmental efficiency. This is due, in particular, to the fact that its economic growth needs to be faster or has directly decreased over time. Some countries with low emissions at the same time as slow growth in GDP or GDP per capita are Bulgaria, Romania, Croatia, Slovakia, Slovenia, Estonia, Greece, Hungary, Portugal and the Czech Republic. They are countries, although they present downward trends, there are cases such as Slovenia or Croatia that, although they are countries with low emissions compared to other countries, these very little throughout the years of the period since they remain practically constant giving to understand that nothing is being proposed to mitigate them and, therefore, eco-efficiency is compromised.

## Conclusions

In conclusion, the results have shown that the founding countries or those that have been part of the European Union for the longest time are the ones that generally have a high eco-efficiency. In this study case period (2012–2020), it can be seen that it was in 2014, there were a greater number of countries with excellent environmental efficiency (16 countries), while 2020 is the year in which the number of countries with excellent eco-efficiency is ten, which may be due to the pandemic caused by SARS-COVID-19. As we explained above, this occurs because although emissions drop and some countries increase their eco-efficiency, most end up reducing them at the cost of a decrease in their economic indicators. This is what we have highlighted throughout this study because for a country to be as eco-efficient as possible, there must be an optimal relationship between its economic and environmental indicators. The global eco-efficiency value for the European Union as a whole is 0.69. This value is lower than in the period between 2005 and 2012, which was 0.78, but it ensures that the European Union is still within the category of medium environmental efficiency. This is because although some countries have improved their environmental efficiency or maintained it, some countries have experienced a decrease in terms of the values they had at the beginning. The decrease in eco-efficiency is mainly because economically, there are still countries that, when it seemed that they were beginning to recover from the 2008 crisis, encountered other problems, such as the case of Greece and the crisis it experienced in 2012.

Between the two methods used to calculate eco-efficiency, it can be seen that the results obtained are quite similar. However, it is always maintained that the calculations made by method 2 are lower than those of method 1 because if a country has high methane emissions, it will be harmed in terms of eco-efficiency, as is the case of Lithuania already mentioned above. These methane emissions must be considered since they are essential to achieving good environmental efficiency. It is necessary to adopt policies that mitigate their emission at the European Union level. In short, the DEA method (or methods) helps to categorize the different countries studied according to their eco-efficiency based on the other data offered by the different official sources, so we can conclude that it is a robust method that allows obtaining fairly precise values for have a broad vision. As we say, it allows for obtaining a general vision. However, it is necessary to compare it with the available data to understand the reason for the resulting eco-efficiency in a specific country. For this reason, it is a method that always invites improvement because the optimum point has not been found to decide what triggers eco-efficiency and, therefore, a method is used that goes further and allows biasing the values (MAM) and making a more appropriate comparison between economic and environmental indicators. This study also provides a computerized methodology, minimizing the error percentage. One of the strengths of the study is the ranking it provides over a long period of time spanning 15 years (2005–2020) which clearly indicates that adapted energy policies in the European Union are beginning to have encouraging results. Despite the latter, it can be said that it is a general classification that opens a horizon for future lines of research:

- Calculate eco-efficiency using other inputs and outputs: combine different inputs, such as natural gas, with desired outputs, such as the employment rate, and unwanted outputs, such as nitrogen and sulfur oxides, which are not used in this research.
- Combination with other methods: this study lays the foundations so that it can be used as a preamble to other studies that use other scenario analysis methodologies (for example, the LMDI method (Logarithmic Mean Divisia Index) or Tapio decoupling in order to combine it and determine if the results obtained are related to each other.

### Supplementary Information

Below is the link to the electronic supplementary material.

Supplementary file1 (XLSX 234 kb)

Supplementary file2 (XLSX 51 kb)

Supplementary file3 (XLSX 60 kb)

## Data Availability

The materials and data used for the development of this research project can be found in various open-access sources such as Eurostat (https://ec.europa.eu/eurostat), the World Bank database (https://www.bancomundial.org/es/home) and by the Intergovernmental Panel on Climate Change (IPCC) (https://www.ipcc.ch) among others.

## Appendix

See Figs. 11, 12, 13 and 14.
